# Potential role of an antimicrobial peptide, KLK in inhibiting lipopolysaccharide-induced macrophage inflammation

**DOI:** 10.1371/journal.pone.0183852

**Published:** 2017-08-29

**Authors:** Pornpimon Jantaruk, Sittiruk Roytrakul, Sutthirat Sitthisak, Duangkamol Kunthalert

**Affiliations:** 1 Department of Microbiology and Parasitology, Faculty of Medical Science, Naresuan University, Phitsanulok, Thailand; 2 Genome Institute, National Center for Genetic Engineering and Biotechnology, National Science and Technology Development Agency, Thailand Science Park, Pathumthani, Thailand; 3 Centre of Excellence in Medical Biotechnology, Faculty of Medical Science, Naresuan University, Phitsanulok, Thailand; Bose Institute, INDIA

## Abstract

Antimicrobial peptides (AMPs) are attractive alternatives to antibiotics. Due to their immune modulatory properties, AMPs are at present emerging as promising agents for controlling inflammatory-mediated diseases. In this study, anti-inflammatory potential of an antimicrobial peptide, KLK (KLKLLLLLKLK) and its analogs was evaluated in lipopolysaccharide (LPS)-induced RAW 264.7 macrophages. The results herein demonstrated that KLK peptide as well as its analogs significantly inhibited the pro-inflammatory mediator nitric oxide (NO), interleukin-1β (IL-1β) and tumor necrosis factor-α (TNF-α) production in LPS-stimulated RAW 264.7 macrophages in dose-dependent manners, and such inhibitory effects were not due to direct cytotoxicity. When considering inhibition potency, KLK among the test peptides exhibited the most effective activity. The inhibitory activity of KLK peptide also extended to include suppression of LPS-induced production of prostaglandin E_2_ (PGE_2_). KLK significantly decreased mRNA and protein expression of inducible nitric oxide synthase (iNOS) and cyclooxygenase-2 (COX-2) as well as mRNA expression of IL-1β and TNF-α. Moreover, KLK inhibited nuclear translocation of nuclear factor-κB (NF-κB) p65 and blocked degradation and phosphorylation of inhibitor of κB (IκB). Taken together, these results suggested that the KLK peptide inhibited inflammatory response through the down-regulation of NF-κB mediated activation in macrophages. Since peptide analogs with different amino acid sequences and arrangement were investigated for their anti-inflammatory activities, the residues/structures required for activity were also discussed. Our findings therefore proved anti-inflammatory potential of the KLK peptide and provide direct evidence for therapeutic application of KLK as a novel anti-inflammatory agent.

## Introduction

Inflammation is a complex process that occurs in the body in response to endo- and exogenous stimuli. Macrophages play a central role in the generation of inflammatory responses by releasing a variety of inflammatory mediators such as nitric oxide (NO), prostaglandin E_2_ (PGE_2_) and pro-inflammatory cytokines including interleukin-1β (IL-1β) and tumor necrosis factor-α (TNF-α)[1]. Although their release serves as a protective response, excessive or dysregulated production of these mediators has been implicated in many chronic inflammatory diseases including rheumatoid arthritis, diabetes, cardiovascular disease, atherosclerosis and cancer [2–4]. Chronic inflammatory-derived diseases are a major public health problem and remain the leading cause of death globally, regardless of national economic status. Effective treatment of chronic inflammation is thus of great importance, and regulation of inflammatory mediator release is potentially beneficial to control severe disease-associated inflammation.

Antimicrobial peptides (AMPs) are evolutionarily conserved small peptides produced by living organisms of all types and considered to be a component of host innate immunity [5]. AMPs have broad-spectrum antimicrobial activity against an array of microbes including bacteria, virus, fungi and certain parasites [6]. More significantly, AMPs can kill multidrug-resistant microorganisms [7] and this ability makes these molecules attractive candidates for antibiotics [8]. Nevertheless, the therapeutic application of AMPs is not limited to their antimicrobial function. AMPs have been documented to perform many other activities, including modulation of the immune response [9,10], anti-angiogenic and anti-tumor activities [11,12]. Indeed, some AMPs have been suggested to have far more potent immunomodulatory activities than antimicrobial functions [13]. Currently, the therapeutic application of AMPs has expanded to involve a number of inflammatory-derived diseases. Since peptides of small size can be metabolically cleaved and rapidly cleared from body, they do not accumulate in specific organs thereby minimizing their toxic side effects [14]. These characteristics make AMPs superior to small molecules, and open a new therapeutic option which is safe and effective for the treatment of inflammatory-mediated diseases.

KLKLLLLLKLK-NH_2_ (KLK) is a synthetic antimicrobial peptide derived from sapecin B, an antibacterial protein of *Sarcophaga peregrina* (flesh fly) [15]. KLK peptide was found to have potent microbicidal activities against *Staphylococcus aureus*, *Escherichia coli*, methicillin-resistant *S*. *aureus* (MRSA) and *Candida albicans* [15]. This peptide also showed significant efficacy in prophylactic treatment of MRSA-infected mice [16]. Moreover, KLK had the ability of activating human neutrophils and U937 monocytes to produce superoxide anions through binding to cell surface calreticulin [17,18]. KLK has also been reported to enhance antigen presentation in mice and act as an effective adjuvant with oligonucleotide-containing deoxyinosine or deoxycytosine [19,20]. These findings suggested immunomodulatory properties of the KLK peptide and raise the possibility of developing this peptide as an immune-modulating agent.

Encouraged by the undesirable toxicity of the widely prescribed non-steroidal anti-inflammatory drugs (NSAIDs) and with the incentive of developing alternative anti-inflammatory agents, the present study evaluated the anti-inflammatory potential of the KLK peptide and its structurally modified and simplified analogs in a lipopolysaccharide (LPS)-stimulated macrophage model. The mode of action responsible for its activity was also investigated.

## Materials and methods

### Peptides

KLK peptide and its truncated analogs listed in Table 1 were synthesized by ChinaPeptides Co., Ltd. (Shanghai, China); the purity of the synthesized peptides was > 95%. Their physico-chemical properties were calculated using APD3: Antimicrobial Peptide Calculator and Predictor (for molecular weight and net charge) [21] and INNOVAGEN Peptide property calculator (for isoelectric point (pI)) [22]. Helical wheel projections and hydrophobicity of all peptides were obtained using Heliquest [23]. These synthetic peptides were dissolved in their vehicle, dimethyl sulfoxide (DMSO; ≥ 99.5%, Sigma, France) and further diluted in culture medium to obtain desired concentrations.

**Table 1:** Physico-chemical properties of KLK and its derivatives.

Peptides	Amino acid sequence	Molecular weight (Da)	Net charge	pI	Hydrophobicity (%)
**KLK**	KLKLLLLLKLK	1322.81	+4	11.15	63.64
**KLK1**	KLKLLLLLKL	1194.66	+3	10.98	70.00
**KLK2**	KLKLLLLLK	1081.50	+3	10.98	66.67
**SSKLK**	CKLKLLLLLKLKC	1529.12	+4	10.42	53.85
**CYCKLK**	KLKLLLLLKLK (N to C cyclization)	1304.81	+4	11.15	63.64

### Cell culture

The murine macrophage RAW 264.7 cell line was obtained from American Type Culture Collection (ATCC; Manassas, VA, USA). Cells were cultured in Dulbecco's Modified Eagle's Medium (DMEM; PAA, Pasching, Austria) supplemented with 10% (v/v) fetal bovine serum (Gibco, South America), 10 mM HEPES (HyClone, Utah, USA), 2 mM L-glutamine (PAA), 100 U/mL penicillin and 100 μg/mL streptomycin (PAA) at 37°C in a humidified atmosphere containing 5% CO_2_.

### Cell viability assay

Cell viability was assessed by the mitochondrial-dependent reduction method of 3-(4,5-dimethylthiazol-2-yl)-2,5-diphenyltetrazolium bromide (MTT) to formazan [24]. Briefly, RAW 264.7 cells were seeded into a 96-well plate (Nunc^™^, Roskilde, Denmark) at a density of 1×10^5^ cells/well and allowed to adhere for 1 h at 37°C in a humidified atmosphere containing 5% CO_2._ The cells were then treated with various concentrations of each peptide (1, 5, 10 and 25 μg/mL) or vehicle. Cells without the test peptide served as untreated control. After 48 h, 20 μL MTT solution (5 mg/mL; Sigma, St. Louis, MO, USA) was added to each well and incubated for another 3 h. After removing the supernatant, 100 μL of DMSO was added to the cells to solubilize the formazan crystals. The optical density (OD) was measured at 540 nm using a microplate reader (Rayto RT-2100C, China). Percentage of cell viability was calculated using an equation: (OD of treated cells/ OD of untreated cells) × 100.

### Determination of NO and PGE_2_ production

RAW 264.7 cells at 1×10^5^ cells/well were stimulated with 1 μg/mL LPS (*Escherichia coli* 0111:B4; Sigma, St. Louis, MO, USA) in the presence or absence of various concentrations of the test peptide and then incubated at 37°C in 5% CO_2_ for 48 h. Indomethacin (10 μM; Sigma, St. Louis, MO, USA) was included as a positive control. Where appropriate, cells were pre-incubated with the test peptide for 1 h, and washed three times with sterile PBS before incubation with LPS (1 μg/mL; Sigma). Culture supernatant was collected and the nitrite (a stable breakdown product of NO) accumulated in culture supernatant was then determined by the Griess reagent system (Promega, Madison, USA). Briefly, 50 μL of culture supernatant was mixed with an equal volume of 1% sulfanilamide in 5% phosphoric acid. After incubation for 10 min at room temperature, 50 μL of 0.1% N-1-napthylethylenediamine dihydrochloride was added and incubated for another 10 min at room temperature. The absorbance was measured at 540 nm using a microplate reader (Rayto RT-2100C, China) and nitrite concentrations were determined using a sodium nitrite standard curve. The PGE_2_ levels in culture medium were quantified by enzyme-immunoassay (R&D Systems, Minneapolis, MN, USA) according to the manufacturer’s instructions.

### Cytokine assays

The pro-inflammatory cytokines (IL-1β and TNF-α)were determined by sandwich enzyme-linked immunosorbent assay (ELISA). RAW 264.7 cells (1×10^5^ cells/well) were seeded into a 96-well microtiter plate (Nunc^™^) and stimulated with LPS (1 μg/mL; Sigma) in the presence or absence of each peptide at different concentrations. After 72 h of incubation, the culture supernatant was collected and cytokine concentrations measured using ELISA MAX^™^ Deluxe Set (BioLegend, San Diego, CA, USA), following the manufacturer’s protocol.

### Reverse transcription polymerase chain reaction (RT-PCR)

RAW 264.7 cells were seeded into a Nunc^™^ 6-well plate (2×10^6^ cells/well) and stimulated with LPS (1 μg/mL; Sigma) in the presence or absence of KLK peptide (5, 10 and 25 μg/mL). After 18 h incubation at 37°C in 5% CO_2_, total RNA was extracted using TRIzol^®^ reagent (Invitrogen) and RNA concentration measured by Nanodrop spectrophotometer (NanoDrop Technologies, USA). cDNA was synthesized from 2 μg of total RNA using Oligo-d(T)_18_ primer and HelixCript^™^ Thermo Reverse Transcriptase kit (NanoHelix, South Korea) according to the manufacturer’s protocol. The resulting cDNA (1 μL) was amplified using the following primers: iNOS forward 5’-TTC-CAG-AAT-CCC-TGG-ACA-AG-3’, reverse 5’-TGG-TCA-AAC-TCT-TGG-GGT-TC-3’; COX-2 forward 5’-AGA-AGG-AAA-TGG-CTG-CAG-AA-3’, reverse 5’-GCT-CGG-CTT-CCAGTA-TTG-AG-3’; IL-1β forward 5’-GGG-CCT-CAA-AGG-AAA-GAA-TC-3’, reverse 5’-TAC-CAG-TTG-GGG-AAC-TCT-GC-3’; TNF-α forward 5’-AGC-CCC-CAG-TCT-GTA-TCC-TT-3’, reverse 5’-CAT-TCG-AGG-CTC-CAG-TGA-AT-3’; GAPDH forward 5’-GAG-TCA-ACG-GAT-TTG-GTC-GT-3’, reverse 5’-GAC-AAG-CTT-CCC-GTT-CTC-AG-3’[25]. The PCR amplification was carried out for 35 cycles at 95°C for 45 s, 60°C for 1 min and 72°C for 45 s (for iNOS and IL-1β), 95°C for 45 s, 65°C for 1 min and 72°C for 45 s (for COX-2), 95°C for 1 min, 63°C for 1 min and 72°C for 1 min (for TNF-α), 95°C for 45 s, 55°C for 1 min and 72°C for 45 s (for GAPDH). The PCR products were separated by electrophoresis on a 1.5% agarose gel and visualized by ethidium bromide staining under UV illumination. Intensities of bands were determined using ImageJ software. Relative expressions were calculated and normalized to the values obtained with the housekeeping GAPDH gene.

### Western blot analysis

RAW 264.7 cells were cultured in a Nunc^TM^ 6-well plate (2×10^6^ cells/well) and stimulated with LPS (1 μg/mL; Sigma) in the presence or absence of KLK peptide. After washing with cold-PBS, the cells were lysed with RIPA lysis buffer (Amresco, OH, USA) containing protease inhibitor cocktails (1X, Amresco). Nuclear and cytosolic proteins were extracted using NE-PER Nuclear and Cytoplasmic Extraction Reagents (Pierce Biotechnology, IL, USA) and a Halt^TM^ protease and phosphatase inhibitor cocktails (Pierce Biotechnology). Protein concentration was determined using a Bradford protein assay kit (Bio-Rad, USA). The extracted proteins (25 μg) were separated electrophoretically by NuPAGE^®^ Novex^®^ 10% Bis-Tris (Invitrogen) and transferred to nitrocellulose membranes (Bio-Rad) with semi-dry transfer system (Bio-Rad). The membranes were blocked with 5% skim milk in TTBS (20 mM Tris, 150 mM NaCl, 0.1% Tween20) at room temperature for 1 h and subsequently incubated overnight with antibodies specific for iNOS (sc-7271, Santa Cruz Biotechnology, USA), COX-2 (sc-1745, Santa Cruz Biotechnology), NF-κB p65 (sc-8008, Santa Cruz Biotechnology), IκB (sc-1643, Santa Cruz Biotechnology) or phospho-IκB (ab-12135, Abcam, USA) and β-actin (ab-170325, Abcam). After washing with TTBS, the membranes were reacted with horseradish peroxidase-conjugated donkey anti-goat IgG (sc-2020, Santa Cruz Biotechnology) or peroxidase-conjugated AffiniPure goat anti-mouse IgG (115-035-003, Jackson ImmunoResearch Laboratories, USA), as appropriate. After 1 h incubation at room temperature, the target proteins were detected by Clarity^™^ Western ECL Substrate (Bio-Rad) according to the manufacturer’s instruction and then captured using an ImageQuant LAS 4000 Biomolecular Imager (GE Healthcare Life Sciences, UK). The relative intensities of the protein bands were measured by ImageJ software and then normalized with β-actin.

### LPS binding assay

The binding of KLK peptide to LPS was determined using a Thermo Scientific™ Pierce™ LAL chromogenic endotoxin quantitation kit. Briefly, 25 μL of the KLK peptide was added in duplicate to 25 μL of *Escherichia coli* 0111:B4 LPS (1.0 EU/mL final concentration) for 5 and 30 min at 37°C, followed by incubation with 50 μL of Limulus Amebocyte Lysate for 10 min at 37°C. Then, 100 μL of the chromogenic substrate; Ac-Ile-Glu-Ala-Arg-p-Nitroaniline was added. After incubation at 37°C for 6 min, the reaction was stopped by the addition of 50 μL of 25% acetic acid and the OD read at 405 nm using a microplate reader. The amount of non-bound LPS was extrapolated from a standard curve, and the percentage of peptide binding to LPS calculated.

### Statistical analysis

Data are presented as mean ± standard deviation (SD) of independent experiments. Statistical analyses were performed with two-tailed Student’s *t*-test using the SPSS version 20 software (SPSS, Chicago, IL, USA). A *P*-value < 0.05 was considered statistically significant.

## Results

### Effect of KLK peptide and its analogs on cell viability

**Fig 1:**
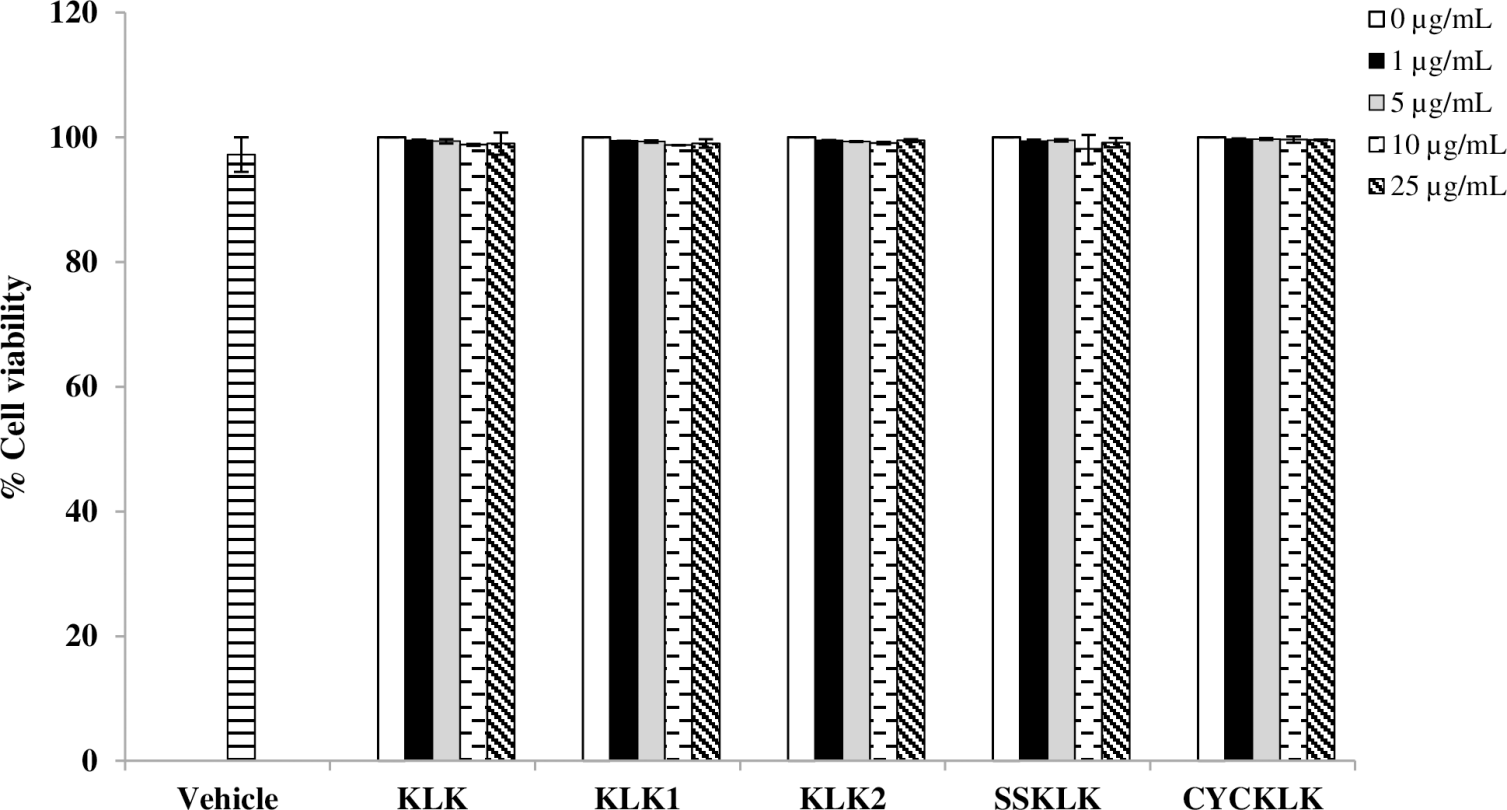
Effect of KLK peptide and its analogs on cell viability. RAW 264.7 cells were treated with various concentrations of each peptide or vehicle for 48 h and cell viability was assessed by an MTT assay. Data are presented as mean ± SD of independent experiments.

Prior to investigating the anti-inflammatory potential of the peptides, their cytotoxicity against RAW 264.7 macrophages was evaluated by an MTT assay. As shown in Fig 1, viability of cells exposed to KLK peptide or its analogs was not significantly changed at any of the peptide concentrations (1–25 μg/mL) compared with the untreated control. MTT assay also showed no difference in cell viability between vehicle and untreated control. These results indicated that all the test peptides at the concentrations examined as well as the vehicle were not cytotoxic to RAW 264.7 macrophage cells.

### Effect of KLK peptide and its analogs on NO production in LPS-stimulated RAW 264.7 cells

**Fig 2:**
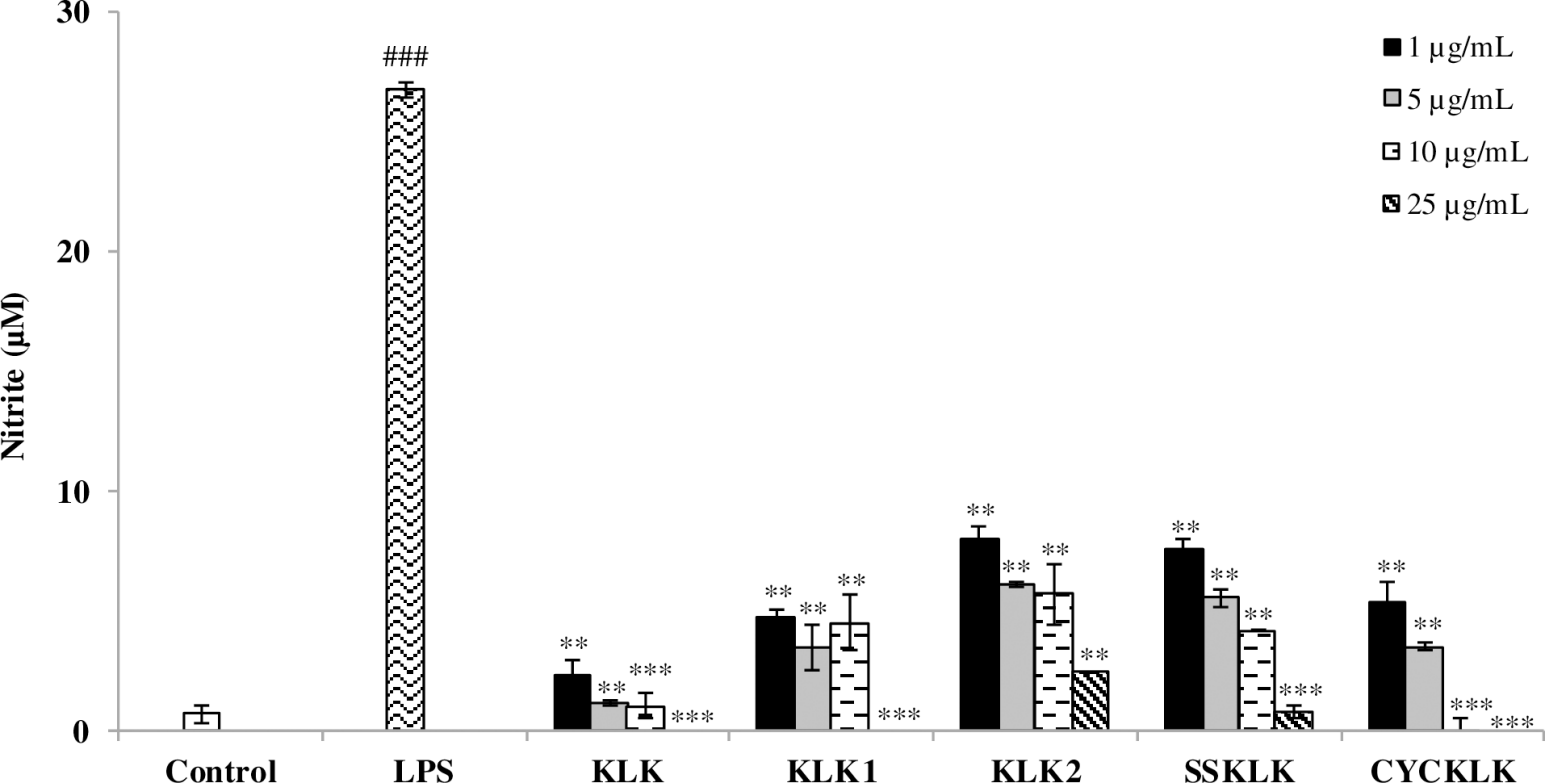
Effects of KLK peptide and its analogs on LPS-stimulated NO production in RAW 264.7 macrophages. Cells were stimulated with LPS (1 μg/mL) in the presence or absence of different concentrations of each peptide for 48 h. The level of nitrite in culture supernatant was measured by Griess reagents. Data are presented as mean ± SD of independent experiments. ###, *P* <0.001 compared with the unstimulated macrophages; **, *P* < 0.01 and ***, *P* < 0.001 compared with the LPS-stimulated macrophage cells.

To assess the potential anti-inflammatory activity of KLK peptide and its analogs, their effect on LPS-stimulated NO production was examined using Griess assay. As presented in Fig 2, nitrite concentration increased markedly in culture supernatant upon LPS stimulation compared with that of the untreated control. However, in the presence of the test peptides, substantial reduction in LPS-stimulated production of NO was observed and the effect appeared to be dose-related. Strong inhibitory effects were apparently detected when cells were exposed to the test peptides, even at the concentration as low as 1 μg/mL. At this concentration, KLK peptide was the most effective with the inhibitory rate of 91.00 ± 2.37%, followed by peptides KLK1, CYCKLK, SSKLK and KLK2 (inhibitory rates of 81.48 ± 0.97%, 79.26 ± 3.17%, 70.77 ± 1.55% and 69.22 ± 1.89%, respectively). It is interesting to note that nearly complete NO inhibition, where the concentration of NO was comparable and not statistically different (*P* > 0.05) from that of the untreated control, was observed in the cells treated with KLK at concentration of 5 μg/mL. This observation was also seen in the cells treated with other KLK analogs but at higher concentrations.

### Effect of KLK peptide and its analogs on pro-inflammatory cytokine production in LPS-stimulated RAW 264.7 cells

**Fig 3:**
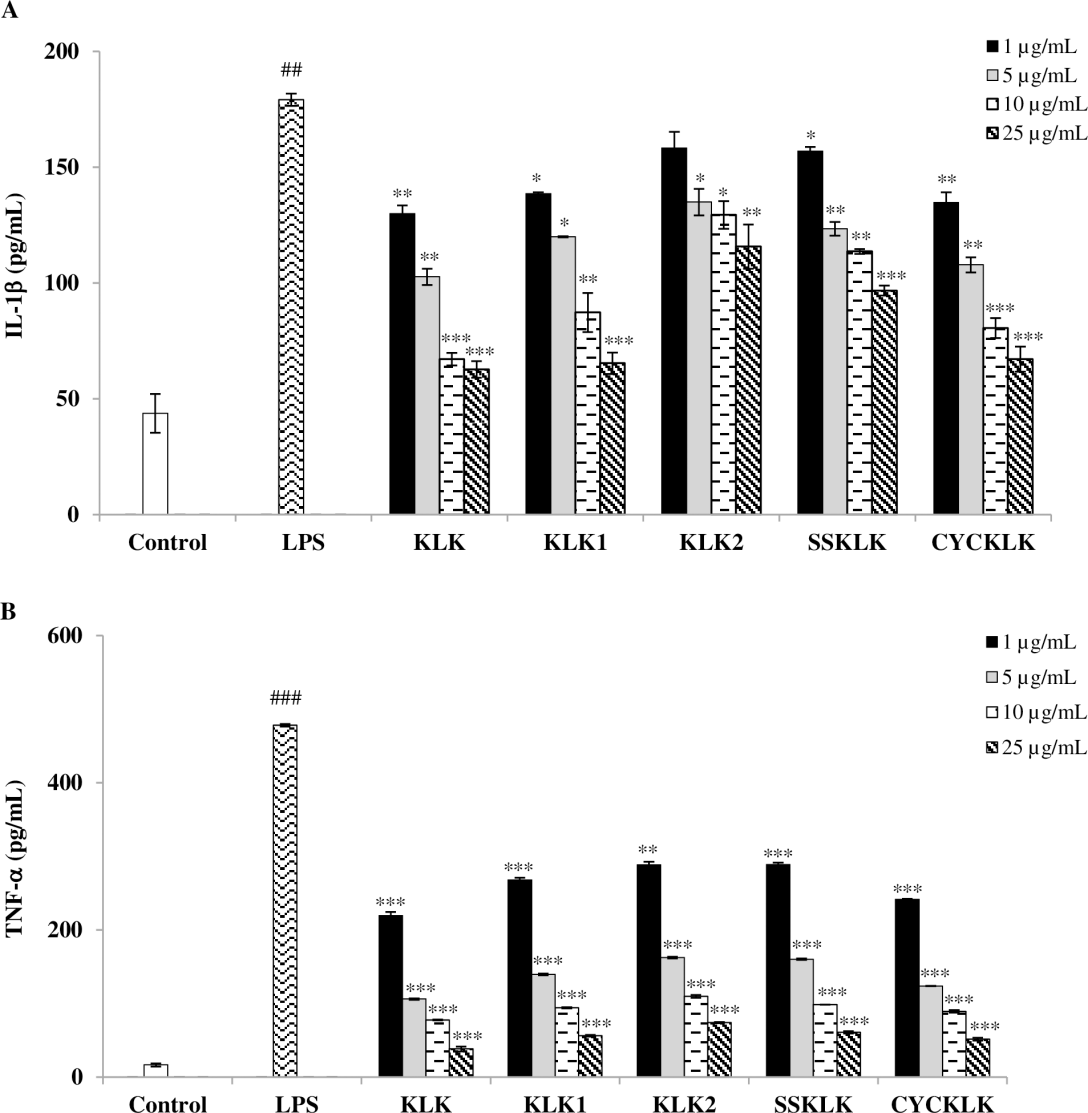
Effects of KLK peptide and its analogs on pro-inflammatory cytokine production in LPS-stimulated RAW 264.7 macrophages. Cells were stimulated with LPS (1 μg/mL) in the presence or absence of different concentrations of individual peptides for 72 h. The levels of IL-1β (A) and TNF-α (B) were evaluated by sandwich ELISA. Data are presented as mean ± SD of independent experiments. ##, *P* <0.01 and ###, *P* <0.001 compared with the unstimulated macrophages; *, *P* < 0.05; **, *P* < 0.01 and *** *P* < 0.001 compared with the LPS-stimulated macrophage cells.

To further evaluate the anti-inflammatory activity of KLK peptide and its analogs, their effects on the production of representative pro-inflammatory cytokines, IL-1β and TNF-α, in LPS-stimulated RAW 264.7 cells were determined by sandwich ELISA. Fig 3 demonstrated that stimulation of RAW 264.7 cells with LPS resulted in increased IL-1β and TNF-α concentrations in culture supernatants, which were significantly reduced by treatment with the test peptides in a concentration-dependent manner compared with the supernatant from cells stimulated with LPS alone. Although all the test peptides exhibited significant inhibitory effects on LPS-stimulated production of IL-1β (Fig 3A) and TNF-α (Fig 3B), their individual potency varied. Among which, KLK peptide exerted the most potent inhibitory activity, with the IC_50_ values of 6.24 ± 0.23 μg/mL and 0.62 ± 0.002 μg/mL for IL-1β and TNF-α, respectively. Decreased IL-1β production to the concentration that was comparable (*P* > 0.05) to the untreated control was also noted with KLK treatment at 10 and 25 μg/mL. Inhibitory activities were less pronounced for peptides CYCKLK, KLK1 and SSKLK; IC_50_ values of 8.83 ± 1.31 μg/mL and 0.91 ± 0.07 μg/mL, 11.03 ± 2.85 μg/mL and 1.35 ± 0.03 μg/mL, and 36.79 ± 1.64 μg/mL and 1.79 ± 0.01 μg/mL, for IL-1β and TNF-α, respectively. KLK2 peptide showed the least inhibitory effect; IC_50_ values > 25 μg/mL and 1.84 ± 0.02 μg/mL, for IL-1β and TNF-α, respectively. According to the results obtained, the KLK peptide was selected for subsequent studies.

### Effect of KLK peptide on PGE_2_ production in LPS-stimulated RAW 264.7 cells

**Fig 4:**
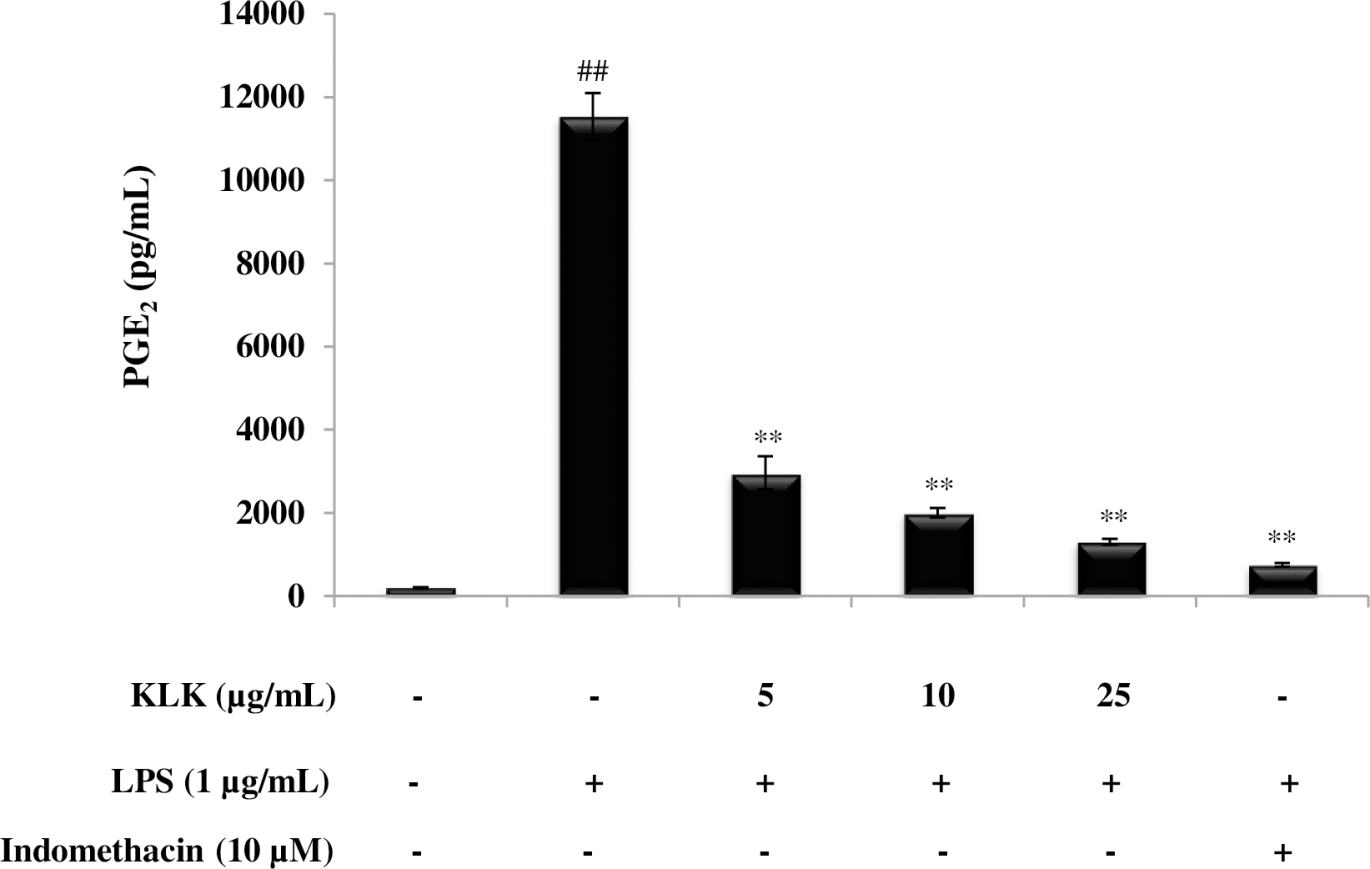
Effect of KLK peptide on LPS-induced PGE_2_ production in RAW 264.7 macrophages. Cells were stimulated with LPS (1 μg/mL) in the presence or absence of KLK peptide (5, 10 and 25 μg/mL) or indomethacin (10 μM) for 48 h. After the incubation, PGE_2_ concentration in culture supernatant was measured by enzyme-immunoassay. Data are presented as mean ± SD of independent experiments. ##, *P* <0.01 compared with the unstimulated macrophages; **, *P* < 0.01 compared with the LPS-stimulated macrophage cells.

PGE_2_ is another inflammatory mediator that plays an important role in the inflammatory response. The involvement of KLK peptide in the production of this mediator was also investigated. As demonstrated in Fig 4, KLK peptide significantly inhibited (*P* < 0.01) LPS-induced PGE_2_ production in a dose-dependent manner. It is noted that KLK peptide at 25 μg/mL inhibited PGE_2_ production by 88.66 ± 0.01%; this was in similar level (*P* > 0.05) to that of a COX-2 inhibitor, indomethacin.

### Effect of KLK peptide on iNOS, COX-2, IL-1β and TNF-α mRNA expression in LPS-stimulated RAW 264.7 cells

**Fig 5:**
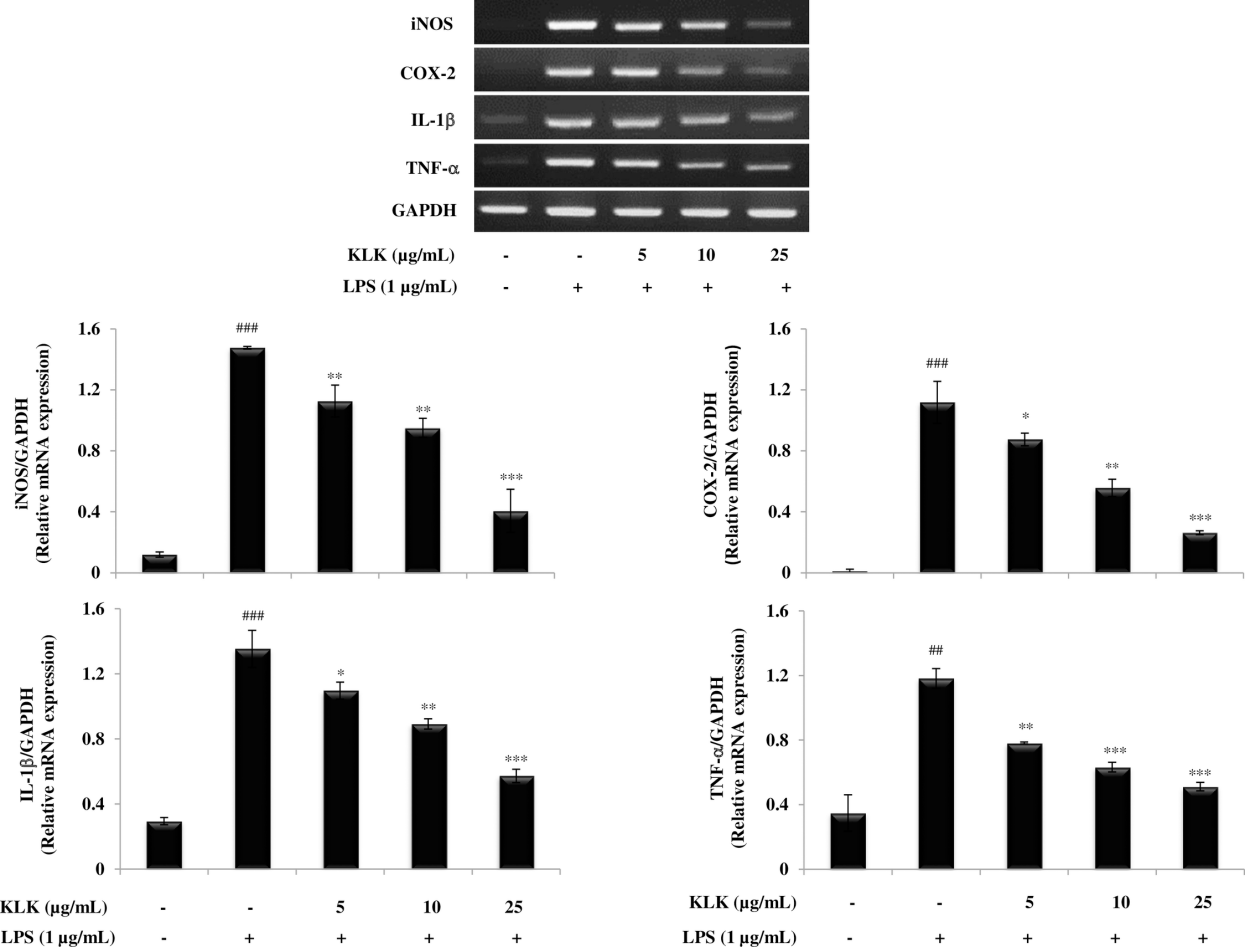
Effects of KLK peptide on iNOS, COX-2, IL-1β and TNF-α mRNA expression in LPS-stimulated RAW 264.7 macrophages. Cells were stimulated with LPS (1 μg/mL) in the presence or absence of different concentrations of KLK peptide for 18 h. Total mRNA was isolated and the mRNA expression of iNOS, COX-2, IL-1β and TNF-α was examined by RT-PCR. Data are expressed as means ± SD of three independent experiments. ##, *P* <0.01 and ###, *P* <0.001 compared with the unstimulated macrophages; *, *P* < 0.05; **, *P* < 0.01 and *** *P* < 0.001 compared with the LPS-stimulated macrophage cells.

To understand whether the KLK peptide affected LPS-induced activation of iNOS, COX-2, IL-1β and TNF-α mRNA expression, a semi-quantitative RT-PCR was performed and the results are shown in Fig 5. Expression of iNOS and COX-2 mRNA was noticeably increased after RAW 264.7 was exposed to LPS (1 μg/mL) for 18 h. However, expression of LPS-induced iNOS and COX-2 mRNA was significantly decreased on treatment with the KLK peptide in a dose-dependent manner. This was also the case for IL-1β and TNF-α mRNA expression in that LPS treatment caused markedly increase in IL-1β and TNF-α mRNA expression, which was significantly inhibited by the increasing concentrations of the KLK peptide.

### Effect of KLK peptide on iNOS and COX-2 protein expression in LPS-stimulated RAW 264.7 cells

**Fig 6:**
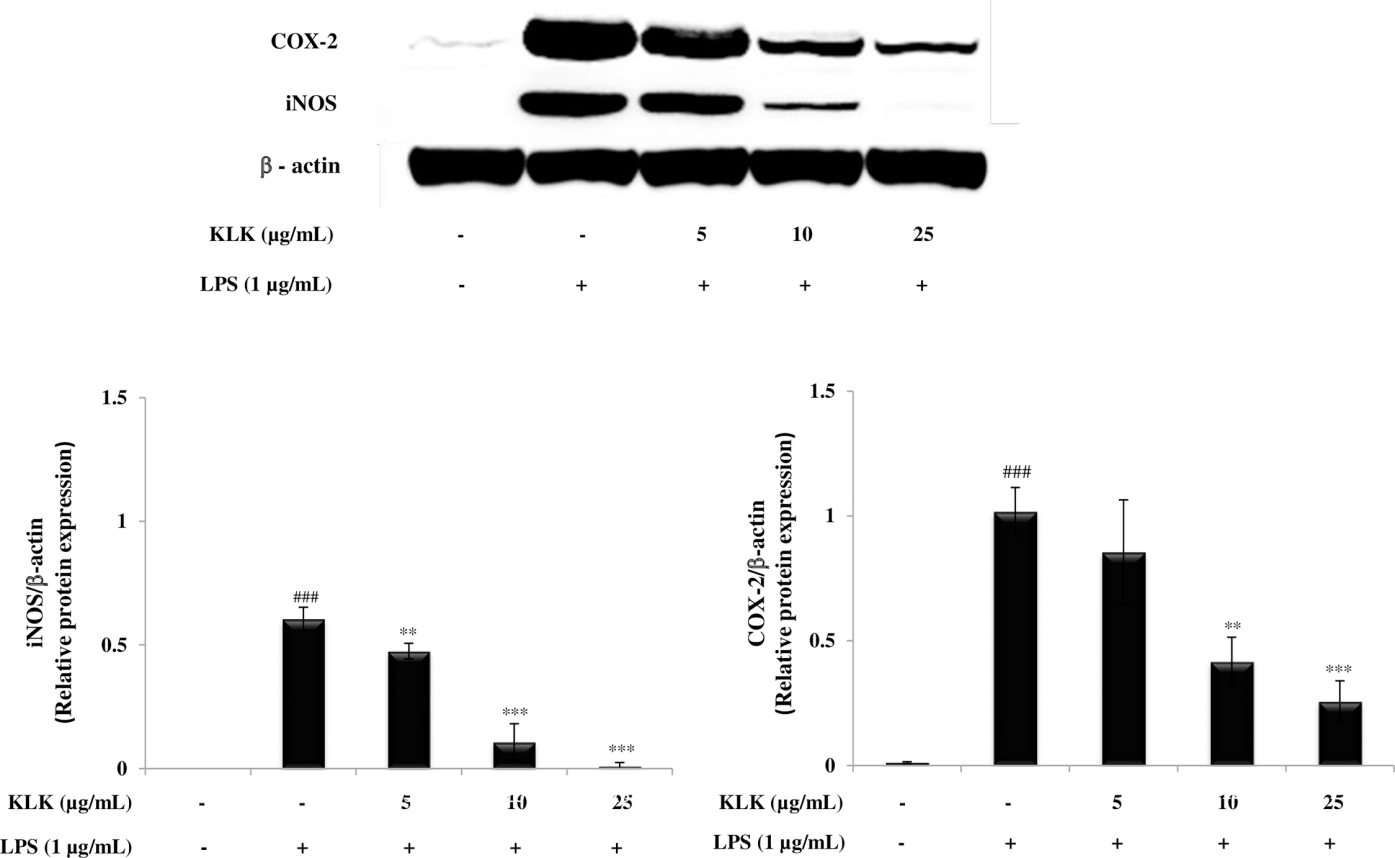
Effects of KLK peptide on iNOS and COX-2 protein expression in LPS-stimulated RAW 264.7 macrophages. Cells were stimulated with LPS (1 μg/mL) in the presence or absence of various concentrations of KLK peptide. After 24 h, the protein expression was determined by Western blotting. Data are presented as mean ± SD of three independent experiments. ###, *P* <0.001 compared with the untreated macrophages; **, *P* < 0.01 and ***, *P* < 0.001 compared with the LPS-stimulated macrophage cells.

Next, to investigate the influence of KLK peptide on expression of inducible nitric oxide synthase (iNOS) and cyclooxygenase (COX)-2 proteins, Western blot analysis was performed. As demonstrated in Fig 6, RAW 264.7 cells treated with LPS displayed a markedly induced expression of iNOS and COX-2 proteins, while the KLK peptide significantly reduced LPS-induced iNOS and COX-2 protein expression in a concentration-dependent manner. Of note, the KLK peptide at 25 μg/mL suppressed the iNOS protein expression to a level close (*P* > 0.05) to that of unstimulated cells.

### Effects of KLK peptide on LPS-induced degradation and phosphorylation of IκB and nuclear translocation of the NF-κB in RAW 264.7 cells

**Fig 7:**
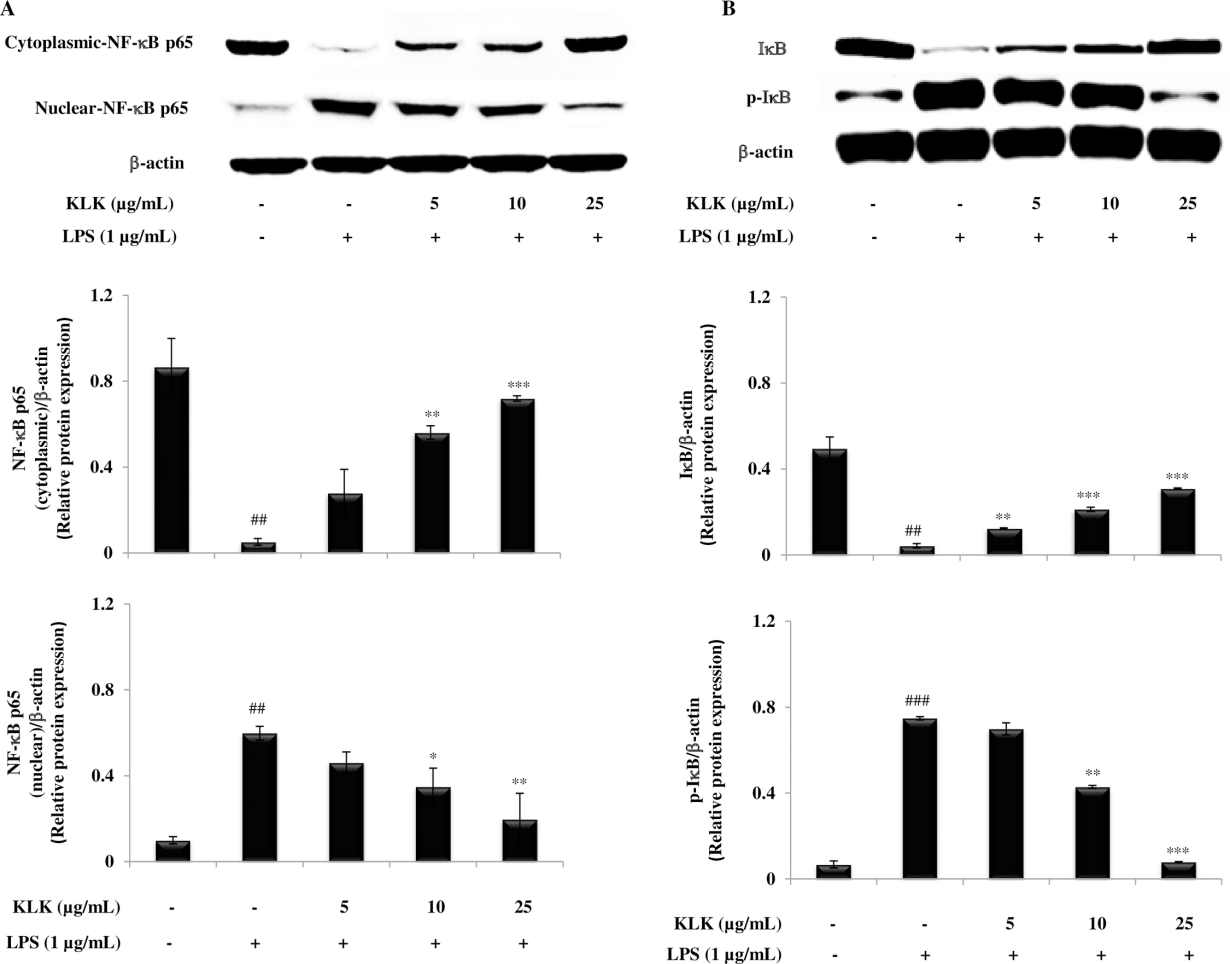
Effects of KLK peptide on LPS-induced activation of NF-κB in RAW 264.7 macrophages. Cells were stimulated with LPS (1 μg/mL) in the presence or absence of KLK peptide. After 30 min incubation, the expression of cytosolic and nuclear protein fractions of NF-κB p65 (A) as well as IκB and its phosphorylated form (B) was detected by Western blotting. The results are presented as mean ± SD of three independent experiments. ##, *P* <0.01 and ###, *P* <0.001 compared with the untreated macrophages; *, *P* < 0.05; **, *P* < 0.01 and ***, *P* < 0.001 compared with the LPS-stimulated macrophage cells.

To better demonstrate the mechanisms involved in the inhibitory effect of the KLK peptide on LPS-induced inflammatory mediators, protein expression of nuclear factor (NF)-κB p65 in the cytosolic and nuclear fractions as well as degradation and phosphorylation of inhibitor of κB (IκB)-α were examined by Western blotting. It was found that NF-κB p65 was less expressed in the cytosol and strongly expressed in the nuclear fraction after the exposure to LPS alone (Fig 7A). However, expression of NF-κB p65 protein in the nuclear fraction was significantly attenuated by treatment with the KLK peptide in a concentration-dependent manner, suggesting that the KLK peptide inhibited the translocation of NF-κB p65 from the cytosol to the nucleus (Fig 7A). Since the nuclear translocation is preceded by the degradation and phosphorylation of IκB-α, the effect of KLK peptide on expression of the IκB-α subunits was also assessed. The results in Fig 7B showed that LPS stimulation dramatically accelerated the degradation of IκB-α and, under the same condition, increased its transformation to p-IκB-α. Treatment with the KLK peptide, however, dose-dependently reversed these effects. It is noticeable that phosphorylation of IκB-α was almost completely blocked with treatment of the KLK peptide at 25 μg/mL.

### Binding studies of KLK peptide to LPS

**Fig 8:**
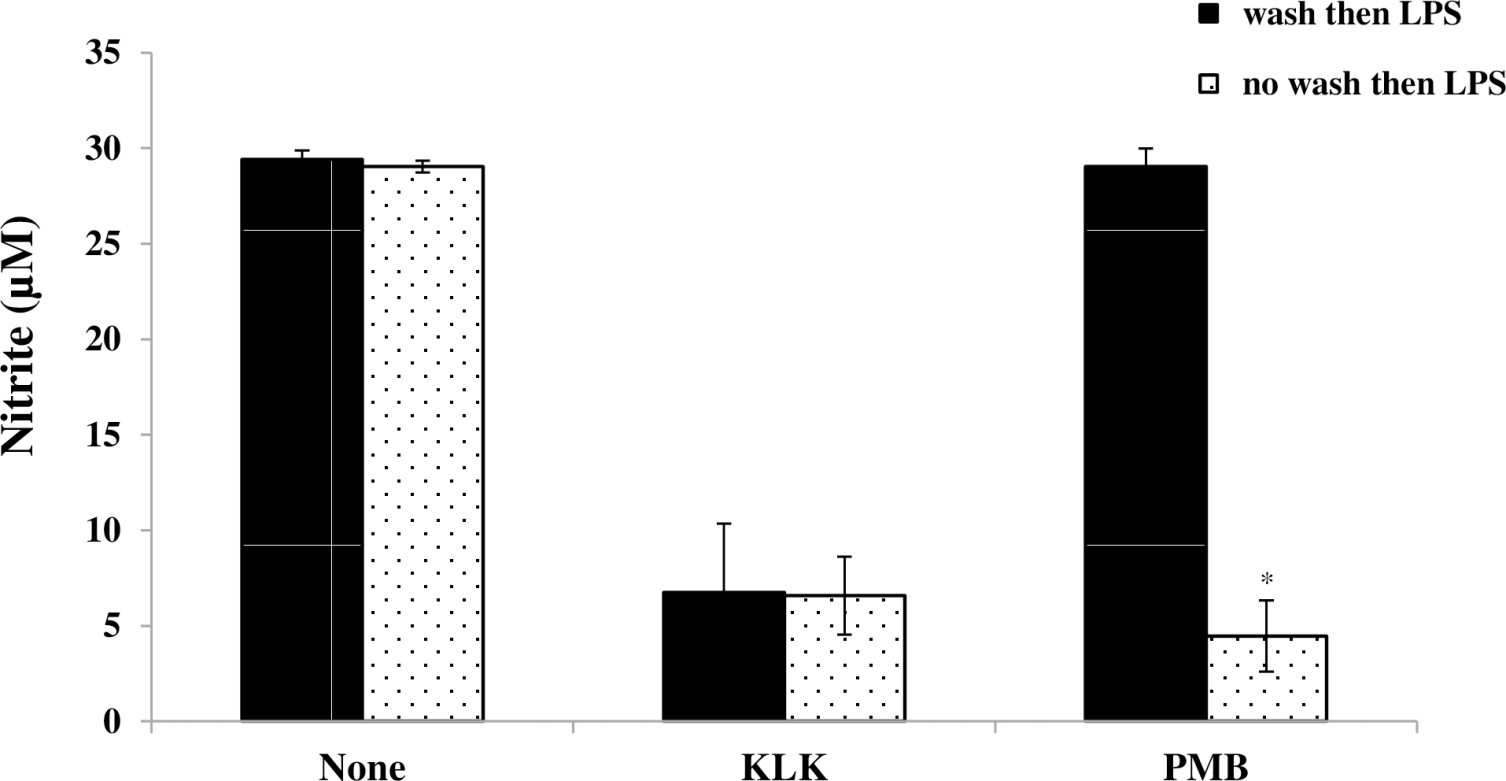
Binding of the KLK peptide to LPS. RAW 264.7 cells were pre-treated in medium containing 10 μg/mL KLK peptide or 10 μg/mL polymyxin B (PMB) for 1 h, washed three times and then stimulated with LPS (1 μg/mL). After 48 hr at 37°C, 5% CO_2_, nitrite from culture supernatant was measured by Griess assay. The results are presented as mean ± SD of independent experiments. *, *P* < 0.05 compared with the respective no wash group.

To investigate whether the ability of KLK peptide to inhibit LPS-induced signaling was due to KLK-LPS binding, experiments were performed in which RAW 264.7 cells were pre-treated with the KLK peptide for 1 h and then thoroughly washed to remove exogenous peptide. The cells were subsequently stimulated with LPS and pro-inflammatory mediator (NO) measured in cell-free supernatant after 48 hr. The results in Fig 8 showed that removal of exogenous KLK peptide prior to LPS stimulation did not alter the ability of the KLK peptide to inhibit the pro-inflammatory effect of LPS. In contrast, the anti-inflammatory effect of a known LPS binding inhibitor PMB was significantly affected by the washing. These results suggested that the action of KLK peptide is not through binding to LPS. An additional assay using a sensitive chromogenic LAL kit was also performed to confirm this observation, and no LPS binding activity of the KLK peptide was evidently observed, even at 25 μg/mL peptide concentration (S1 Table).

## Discussion

This study investigated the action of KLK peptide and its analogs on LPS-induced macrophage inflammation. The results herein showed that KLK peptide as well as its analogs significantly inhibited the pro-inflammatory mediator NO, IL-1β and TNF-α secretion induced by LPS in RAW 264.7 macrophage cells, and such inhibitory activities were not related to direct cellular cytotoxicity. Of the peptides evaluated, KLK peptide appeared to be the most effective; the greatest inhibition potency toward LPS-induced production of NO, IL-1β and TNF-α was clearly seen upon treatment with the KLK peptide. Notably, reduced NO and IL-1β production to the concentrations comparable to that of the untreated control was evident, suggesting the capability of the KLK peptide to restore inflammatory phase to a normal state. KLK peptide also suppressed PGE_2_ production in LPS-stimulated macrophages, and this further strengthened the powerful inhibitory action of the KLK peptide. Since NO, IL-1β, TNF-α and PGE_2_ are key mediators regulating inflammation, and that the KLK peptide inhibited all these crucial inflammatory mediators, our results therefore demonstrate the anti-inflammatory potential of the KLK peptide.

Principally, the inflammatory mediators are regulated primarily at the mRNA level via the involvement of transcription factors, in particular NF-κB [26]. In the resting state, NF-κB exists in the cytoplasm as an inactive heterodimer (p50 and p65) and binds to IκB. Upon stimulation with specific stimuli, most notably LPS, IκB proteins are phosphorylated by IκB kinases (IKKs) and dissociated from NF-κB complexes [27]. The resulting free NF-κB subsequently translocates from cytosol to the nucleus, where it binds to cognate DNA binding sites in the promoter regions of target genes, and activates the transcription of inflammatory mediators such as iNOS, COX-2, IL-1β and TNF-α [27]. This study demonstrated that the KLK peptide inhibited nuclear translocation of NF-κB p65 (Fig 7A) and blocked degradation and phosphorylation of IκB-α (Fig 7B). Also, decreased iNOS, COX-2, IL-1β and TNF-α mRNA expression was observed after treatment with the KLK peptide (Fig 5), and this correlated directly with those of protein expression (Fig 6). Collectively, our results verified that the inhibitory activity in LPS-induced inflammatory mediator NO, IL-1β, TNF-α and PGE_2_ production by KLK peptide is mediated through the inhibition of NF-κB activity. Owing to its important role in regulating inflammatory responses, NF-κB has been an emerging target for controlling inflammation [28,29]. In this context, the KLK peptide would represent a promising therapeutic for treatment of inflammatory diseases involving NF-κB activation.

**Fig 9:**
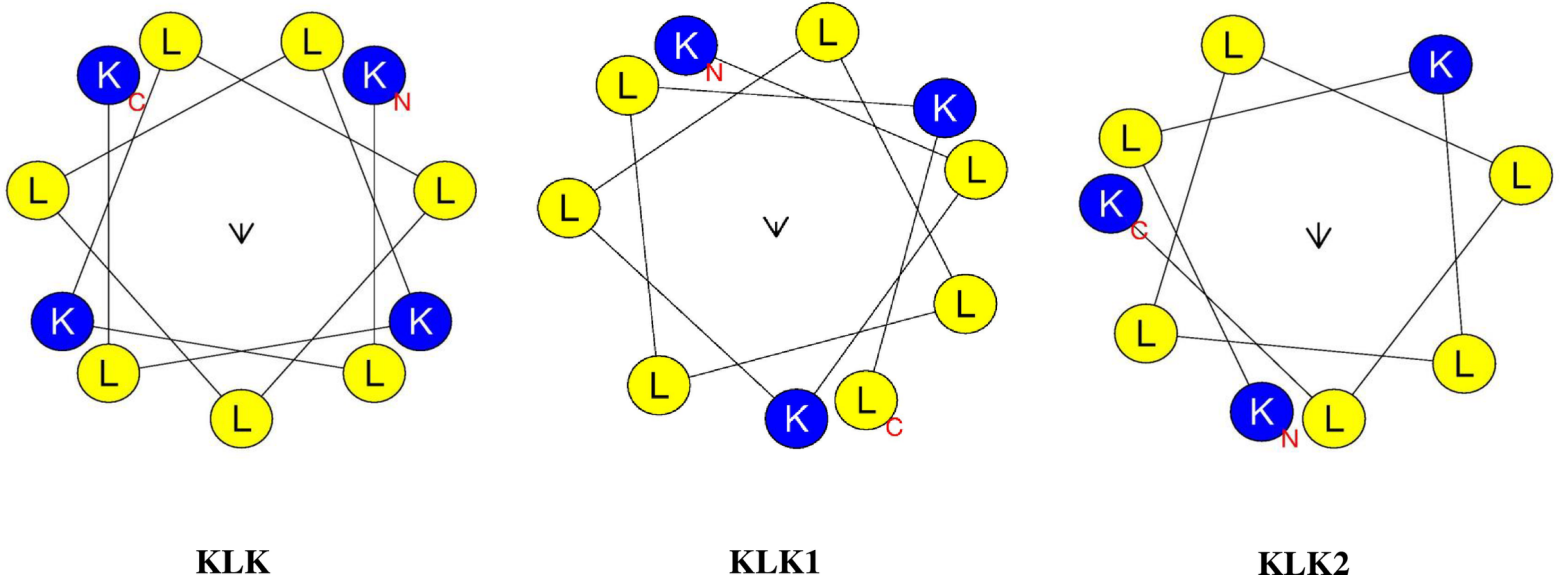
Helical wheel diagram of KLK, KLK1 and KLK2 peptides. The helical wheel projection was performed using online program of the HeliQuest: http://heliquest.ipmc.cnrs.fr [23]. The yellow color represents hydrophobic amino acids and blue color represents the basic residues.

Many AMPs exert their anti-inflammatory effects through LPS-neutralizing activity [30,31]. The present results for the KLK peptide, however, indicated that the observed anti-inflammatory activity is not simply due to LPS neutralization, as removal of exogenous KLK peptide prior to LPS stimulation did not abolish its inhibitory activity, and the LAL assay did not show any evidence activity for LPS-binding after incubation of KLK with LPS. It has been reported that the LPS-binding activity of AMPs depends mainly on net positive charge and hydrophobicity [32]. The net charge of KLK peptide is +4, and helical wheel projection of this peptide showed dispersed, interrupted hydrophobic amino acids (Fig 9). The fact that the hydrophobic face comprising a minimum of 5 uninterrupted hydrophobic amino acid residues is necessary for binding of the negatively charged LPS [33–35], we propose that the lack of LPS-binding activity observed in this study might be related to the dispersity of hydrophobic amino acids in the KLK peptide. In addition to non-LPS neutralization, our results from pre-incubation of peptide and removal of excess unbound peptide also suggested that KLK peptide could act via blocking the TLR4/LPS interaction. Another possibility is that the KLK peptide may penetrate macrophages and subsequently interfere with signaling molecules in the inflammatory pathway to inhibit inflammation. A range of AMPs have been recognized as cell-penetrating peptides and have also been reported to be capable of inhibiting NF-κB signaling and suppressing inflammation [36,37]. Further investigations are required to verify these possible mechanisms of action. Nevertheless, our results would provide a unique property of a peptide with anti-inflammatory potential, and support previous observation that the ability of peptides to block LPS-induced responses does not rely exclusively on the ability to bind to LPS [37,38].

In the present study, KLK peptide and analogs were evaluated for their anti-inflammatory activities. Assessment of anti-inflammatory activity through a series of peptides with different amino acid sequences and arrangement would provide an understanding of the residues/structures required for such activity. Although all the peptides in this study exhibited anti-inflammatory activities, varied potency among individuals was obviously seen. The KLK peptide exerted the strongest anti-inflammatory activity, while its C-terminally truncated forms (KLK1 and KLK2) showed a reduction in activity. Of particular note, a dramatic decrease in inhibitory activity was demonstrated with the KLK2 peptide. Compared to the parent KLK peptide, KLK1 and KLK2 peptides lacked lysine (K), and leucine-lysine (LK) amino acid residues, respectively. When considering the structure of the peptides, helical wheel projection also revealed that the KLK peptide, but not its truncated forms, has helical symmetry (Fig 9). Taken together, these observations indicated the importance of C-terminal L and K residues for efficient anti-inflammatory action, and their presence in the peptide sequence generates a structural arrangement most favorable for inhibitory activity. Consistently, the importance of the C-terminal KLK motif in the KLK peptide has also been reported for other biological activities including anti-microbial activity and neutrophil activation [15,17]. To further determine whether structural modification such as disulfide bonds and cyclization affected the anti-inflammatory activity, the SSKLK (cysteine residues added to both N and C-terminal ends) and CYCKLK peptides were additionally synthesized. Disulfide bonds and cyclization have been described to be involved in bioactivities of numerous peptides [39–42]. Although SSKLK and CYCKLK peptides elicited anti-inflammatory activity, these two analogs had far less activity than the parent KLK peptide, suggesting that such structural changes may not be essential for improvement of the inhibitory activity. In light of these observations, our findings are of significance, providing valuable evidence for peptide design and development as future therapeutics.

## Conclusions

Our results provide the first evidence that the KLK peptide exerted potent anti-inflammatory activity by efficiently inhibiting the crucial inflammatory mediators in LPS-stimulated macrophages without exhibiting cytotoxicity. These inhibitory effects involved the down-regulation of the NF-κB activation pathway. All these properties make the KLK peptide a promising molecule for the development of a novel anti-inflammatory agent. Since the KLK peptide possesses antimicrobial activity, this peptide would also be advantageous for the treatment of infectious inflammation.

## Supporting information

S1 Table LPS binding activity of the KLK peptide. (DOCX)

S1 File The primary data underlying our results. (DOCX)

## References

[1] Fujiwara N, Kobayashi K. Macrophages in inflammation. Curr Drug Targets Inflamm Allergy. 2005;4(3):281–6. 16101534 10.2174/1568010054022024

[2] Udalova IA, Mantovani A, Feldmann M. Macrophage heterogeneity in the context of rheumatoid arthritis. Nat Rev Rheumatol. 2016;12:472–85. doi: 10.1038/nrrheum.2016.91 27383913 10.1038/nrrheum.2016.91

[3] Cochain C, Zernecke A. Macrophages in vascular inflammation and atherosclerosis. Pflugers Arch. 2017;469 (3–4):485–99. doi: 10.1007/s00424-017-1941-y 28168325 10.1007/s00424-017-1941-y

[4] Zhang Q, Zhu B, Li Y. Resolution of cancer-promoting inflammation: A new approach for anticancer therapy. Front Immunol. 2017;8:71 doi: 10.3389/fimmu.2017.00071 28210259 10.3389/fimmu.2017.00071 PMC5288347

[5] Pinheiro da Silva F, Machado MC. Antimicrobial peptides: clinical relevance and therapeutic implications. Peptides. 2012;36(2):308–14. doi: 10.1016/j.peptides.2012.05.014 22659161 10.1016/j.peptides.2012.05.014

[6] Kang H-K, Kim C, Seo CH, Park Y. The therapeutic applications of antimicrobial peptides (AMPs): a patent review. J Microbiol. 2017;55(1):1–12. doi: 10.1007/s12275-017-6452-1 28035594 10.1007/s12275-017-6452-1

[7] Tsai WC, Zhuang ZJ, Lin CY, Chen WJ. Novel antimicrobial peptides with promising activity against multidrug resistant *Salmonella enterica* serovar Choleraesuis and its stress response mechanism. J Appl Microbiol. 2016;121(4):952–65. doi: 10.1111/jam.13203 27280957 10.1111/jam.13203

[8] Peters BM, Shirtliff ME, Jabra-Rizk MA. Antimicrobial peptides: primeval molecules or future drugs? PLoS Pathog. 2010;6(10):e1001067 doi: 10.1371/journal.ppat.1001067 21060861 10.1371/journal.ppat.1001067 PMC2965748

[9] Fabisiak A, Murawska N, Fichna J. LL-37: Cathelicidin-related antimicrobial peptide with pleiotropic activity. Pharmacol Rep. 2016;68(4):802–8. doi: 10.1016/j.pharep.2016.03.015 27117377 10.1016/j.pharep.2016.03.015

[10] Scott MG, Hancock RE. Cationic antimicrobial peptides and their multifunctional role in the immune system. Crit Rev Immunol. 2000;20(5):407–31. 11145218

[11] Jung HJ, Kim Y, Lee HB, Kwon HJ. Antiangiogenic activity of the lipophilic antimicrobial peptides from an endophytic bacterial strain isolated from red pepper leaf. Mol Cells. 2015;38(3):273–8. doi: 10.14348/molcells.2015.2320 25556370 10.14348/molcells.2015.2320 PMC4363728

[12] Chu HL, Yip BS, Chen KH, Yu HY, Chih YH, Cheng HT, et al Novel antimicrobial peptides with high anticancer activity and selectivity. PLoS One. 2015;10(5):e0126390 doi: 10.1371/journal.pone.0126390 25970292 10.1371/journal.pone.0126390 PMC4430538

[13] Scott MG, Davidson DJ, Gold MR, Bowdish D, Hancock RE. The human antimicrobial peptide LL-37 is a multifunctional modulator of innate immune responses. J Immunol. 2002;169(7):3883–91. 12244186 10.4049/jimmunol.169.7.3883

[14] Sun L. Peptide-based drug development. Mod Chem appl. 2013;1:e103.

[15] Alvarez-Bravo J, Kurata S, Natori S. Novel synthetic antimicrobial peptides effective against methicillin-resistant *Staphylococcus aureus*. Biochem J. 1994;302(Pt 2):535–8. 8093007 10.1042/bj3020535 PMC1137260

[16] Nakajima Y, Alvarez-Bravo J, Cho J-H, Homma K-I, Kanegasaki S, Natori S. Chemotherapeutic activity of synthetic antimicrobial peptides: correlation between chemotherapeutic activity and neutrophil-activating activity. FEBS Letters. 1997;415(1):64–6. 9326370 10.1016/s0014-5793(97)01101-0

[17] Cho J-H, Homma KI, Kanegasaki S, Natori S. Activation of human neutrophils by a synthetic anti-microbial peptide, KLKLLLLLKLK-NH_2_, via cell surface calreticulin. Eur J Biochem. 1999;266:878–85. 10583381 10.1046/j.1432-1327.1999.00920.x

[18] Cho JH, Homma KJ, Kanegasaki S, Natori S. Activation of human monocyte cell line U937 via cell surface calreticulin. Cell Stress Chaperones. 2001;6(2):148–52. 11599576 10.1379/1466-1268(2001)006<0148:aohmcl>2.0.co;2 PMC434392

[19] Fritz JH, Brunner S, Birnstiel ML, Buschle M, von Gabain A, Mattner F, et al The artificial antimicrobial peptide KLKLLLLLKLK induces predominantly a Th2-type immune response to co-injected antigens. Vaccine. 2004;22:3274–84. doi: 10.1016/j.vaccine.2004.03.007 15308350 10.1016/j.vaccine.2004.03.007

[20] Schellack C, Prinz K, Egyed A, Fritz JH, Wittmann B, Ginzler M, et al IC31, a novel adjuvant signaling via TLR9, induces potent cellular and humoral immune responses. Vaccine. 2006;24:5461–72. doi: 10.1016/j.vaccine.2006.03.071 16678312 10.1016/j.vaccine.2006.03.071

[21] Wang G, Li X, Wang Z. APD3: the antimicrobial peptide database as a tool for research and education. Nucleic Acids Res. 2016;44:D1087–D93. doi: 10.1093/nar/gkv1278 26602694 10.1093/nar/gkv1278 PMC4702905

[22] Lear S, Cobb SL. Pep-Calc.com: a set of web utilities for the calculation of peptide and peptoid properties and automatic mass spectral peak assignment. J Comput Aided Mol Des. 2016;30:271–7. doi: 10.1007/s10822-016-9902-7 26909892 10.1007/s10822-016-9902-7 PMC4801989

[23] Gautier R, Douguet D, Antonny B, Drin G. HELIQUEST: a web server to screen sequences with specific α-helical properties. Bioinformatics. 2008;24(8):2101–2. 18662927 10.1093/bioinformatics/btn392

[24] Mosmann T. Rapid colorimetric assay for cellular growth and survival: application to proliferation and cytotoxicity assays. J Immunol Methods. 1983;65(1–2):55–63. 6606682 10.1016/0022-1759(83)90303-4

[25] Cho Y-S, Lee S-H, Kim S-W, Ahn C-B, Je J-Y. Aminoethyl-chitosan inhibits LPS-induced inflammatory mediators, iNOS and COX-2 expression in RAW264.7 mouse macrophages. Process Biochem. 2011;46(2):465–70.

[26] Lawrence T, Fong C. The resolution of inflammation: anti-inflammatory roles for NF-kappaB. Int J Biochem Cell Biol. 2010;42(4):519–23. doi: 10.1016/j.biocel.2009.12.016 20026420 10.1016/j.biocel.2009.12.016

[27] Häcker H, Karin M. Regulation and function of IKK and IKK-related kinases. Sci STKE. 2006;2006(357):re13 doi: 10.1126/stke.3572006re13 17047224 10.1126/stke.3572006re13

[28] Wan F, Lenardo MJ. The nuclear signaling of NF-κB–Current knowledge, new insights, and future perspectives. Cell Res. 2010;20(1):24–33. doi: 10.1038/cr.2009.137 19997086 10.1038/cr.2009.137 PMC3052775

[29] Sethi G, Tergaonkar V. Potential pharmacological control of the NF-κB pathway. Trends Pharmacol Sci. 2009;30(6):313–21. doi: 10.1016/j.tips.2009.03.004 19446347 10.1016/j.tips.2009.03.004

[30] Scott A, Weldon S, Buchanan PJ, Schock B, Ernst RK, McAuley DF, et al Evaluation of the ability of LL-37 to neutralise LPS *in vitro* and *ex vivo*. PLoS One. 2011;6(10):e26525 doi: 10.1371/journal.pone.0026525 22028895 10.1371/journal.pone.0026525 PMC3196584

[31] Kim JK, Lee E, Shin S, Jeong KW, Lee JY, Bae SY, et al Structure and function of papiliocin with antimicrobial and anti-inflammatory activities isolated from the swallowtail butterfly, *Papilio xuthus*. J Biol Chem. 2011;286(48):41296–311. doi: 10.1074/jbc.M111.269225 21965682 10.1074/jbc.M111.269225 PMC3308842

[32] Rosenfeld Y, Lev N, Shai Y. Effect of the hydrophobicity to net positive charge ratio on antibacterial and anti-endotoxin activities of structurally similar antimicrobial peptides. Biochemistry. 2010;49(5):853–61. doi: 10.1021/bi900724x 20058937 10.1021/bi900724x

[33] Khara JS, Obuobi S, Wang Y, Hamilton MS, Robertson BD, Newton SM, et al Disruption of drug-resistant biofilms using de novo designed short a-helical antimicrobial peptides with idealized facial amphiphilicity. Acta Biomater. 2017;57:103–14. doi: 10.1016/j.actbio.2017.04.032 28457962 10.1016/j.actbio.2017.04.032

[34] Nagaoka I, Hirota S, Niyonsaba F, Hirata M, Adachi Y, Tamura H, et al Augmentation of the lipopolysaccharide-neutralizing activities of human cathelicidin CAP18/LL-37-derived antimicrobial peptides by replacement with hydrophobic and cationic amino acid residues. Clin Diagn Lab Immunol. 2002;9(5):972–82. doi: 10.1128/CDLI.9.5.972-982.2002 12204946 10.1128/CDLI.9.5.972-982.2002 PMC120071

[35] Cao L, Dai C, Li Z, Fan Z, Song Y, Wu Y, et al Antibacterial activity and mechanism of a scorpion venom peptide derivative *in vitro* and *in vivo*. PLoS ONE. 2012;7(7):e40135 doi: 10.1371/journal.pone.0040135 22792229 10.1371/journal.pone.0040135 PMC3390344

[36] Wang YF, Xu X, Fan X, Zhang C, Wei Q, Wang X, et al A cell-penetrating peptide suppresses inflammation by inhibiting NF-κB signaling. Mol Ther. 2011;19(10):1849–57. doi: 10.1038/mt.2011.82 21556052 10.1038/mt.2011.82 PMC3188757

[37] Lee JY, Suh JS, Kim JM, Kim JH, Park HJ, Park YJ, et al Identification of a cell-penetrating peptide domain from human beta-defensin 3 and characterization of its anti-inflammatory activity. Int J Nanomedicine. 2015;10:5423–34. doi: 10.2147/IJN.S90014 26347021 10.2147/IJN.S90014 PMC4554392

[38] Shim D-W, Heo K-H, Kim Y-K, Sim E-J, Kang T-B, Choi J-W, et al Anti-inflammatory action of an antimicrobial model peptide that suppresses the TRIF-dependent signaling pathway via inhibition of toll-like receptor 4 endocytosis in lipopolysaccharide-stimulated macrophages. PLoS One. 2015;10(5):e0126871 doi: 10.1371/journal.pone.0126871 26017270 10.1371/journal.pone.0126871 PMC4446091

[39] Yousif A, Minopoli M, Bifulco K, Ingangi V, Di Carluccio G, Merlino F, et al Cyclization of the urokinase receptor-derived ser-arg-ser-arg-tyr peptide generates a potent inhibitor of trans-endothelial migration of monocytes. PLoS One. 2015;10(5):e0126172 doi: 10.1371/journal.pone.0126172 25938482 10.1371/journal.pone.0126172 PMC4418665

[40] Chan LY, Zhang VM, Huang YH, Waters NC, Bansal PS, Craik DJ, et al Cyclization of the antimicrobial peptide gomesin with native chemical ligation: influences on stability and bioactivity. Chembiochem. 2013;14(5):617–24. doi: 10.1002/cbic.201300034 23426877 10.1002/cbic.201300034

[41] Gupta K, Kotian A, Subramanian H, Daniell H, Ali H. Activation of human mast cells by retrocyclin and protegrin highlight their immunomodulatory and antimicrobial properties. Oncotarget. 2015;6(30):28573–87. doi: 10.18632/oncotarget.5611 26378047 10.18632/oncotarget.5611 PMC4745678

[42] Lee J, Lee D, Choi H, Kim HH, Kim H, Hwang JS, et al Structure-activity relationships of the intramolecular disulfide bonds in coprisin, a defensin from the dung beetle. BMB Rep. 2014;47(11):625–30. doi: 10.5483/BMBRep.2014.47.11.262 24393527 10.5483/BMBRep.2014.47.11.262 PMC4281341

