# Supplementary material for: Potential role of an antimicrobial peptide, KLK in inhibiting lipopolysaccharide-induced macrophage inflammation
Source: PLoS One. 2017 Aug 29;12(8):e0183852. doi: 10.1371/journal.pone.0183852 (PMC5574609; doi:10.1371/journal.pone.0183852)
Supplement: S1 Table — (DOCX) [file pone.0183852.s001.docx]

| Peptide concentration (μg/mL) | % peptide binding to LPS | |
| --- | --- | --- |
|  | 5 min-incubation | 30 min-incubation |
| 5 | 2.03 | 0.00 |
| 10 | 12.97 | 0.36 |
| 25 | 0.00 | 2.60 |
