# Supplementary material for: Potential role of an antimicrobial peptide, KLK in inhibiting lipopolysaccharide-induced macrophage inflammation
Source: PLoS One. 2017 Aug 29;12(8):e0183852. doi: 10.1371/journal.pone.0183852 (PMC5574609; doi:10.1371/journal.pone.0183852)
Supplement: S1 File — (DOCX) [file pone.0183852.s002.docx]

**Fig 1.** **Effect of KLK peptide and its analogs on cell viability**

| **% Cell viability** | | | | | |
| --- | --- | --- | --- | --- | --- |
|  | **concentration** | **exp. 1** | **exp. 2** | **x̄** | **sd** |
| **Vehicle** |  | 97.58706 | 96.87500 | 97.23103 | 0.50350 |
| **% Cell viability** | | | | | |
| **peptide** | **concentrations** | **exp. 1** | **exp. 2** | **x̄** | **sd** |
| **KLK** | 0 µg/mL | 100.0000 | 100.0000 | 100.0000 | 0.00000 |
|  | 1 µg/mL | 99.37549 | 99.61801 | 99.49675 | 0.17149 |
|  | 5 µg/mL | 99.59016 | 99.13516 | 99.36266 | 0.32173 |
|  | 10 µg/mL | 98.91114 | 98.70968 | 98.81041 | 0.14245 |
|  | 25 µg/mL | 100.23490 | 97.74421 | 98.98957 | 1.76120 |
| **% Cell viability** | | | | | |
| **peptide** | **concentrations** | **exp. 1** | **exp. 2** | **x̄** | **sd** |
| **KLK1** | 0 µg/mL | 100.0000 | 100.0000 | 100.0000 | 0.00000 |
|  | 1 µg/mL | 99.45355 | 99.41697 | 99.43526 | 0.025869 |
|  | 5 µg/mL | 99.47307 | 99.19549 | 99.33428 | 0.196274 |
|  | 10 µg/mL | 98.71670 | 98.77016 | 98.74343 | 0.037801 |
|  | 25 µg/mL | 99.49099 | 98.50957 | 99.00028 | 0.693974 |
| **% Cell viability** | | | | | |
| **peptide** | **concentrations** | **exp. 1** | **exp. 2** | **x̄** | **sd** |
| **KLK2** | 0 µg/mL | 100.0000 | 100.0000 | 100.0000 | 0.00000 |
|  | 1 µg/mL | 99.55113 | 99.49739 | 99.52426 | 0.03800 |
|  | 5 µg/mL | 99.29742 | 99.37651 | 99.33697 | 0.05592 |
|  | 10 µg/mL | 99.22224 | 98.93145 | 99.07685 | 0.20562 |
|  | 25 µg/mL | 99.35395 | 99.63746 | 99.49571 | 0.20047 |
| **% Cell viability** | | | | | |
| **peptide** | **concentrations** | **exp. 1** | **exp. 2** | **x̄** | **sd** |
| **SSKLK** | 0 µg/mL | 100.0000 | 100.0000 | 100.0000 | 0.00000 |
|  | 1 µg/mL | 99.55113 | 99.17571 | 99.36342 | 0.265461 |
|  | 5 µg/mL | 99.66823 | 99.37651 | 99.52237 | 0.206277 |
|  | 10 µg/mL | 96.50010 | 99.73790 | 98.11900 | 2.289475 |
|  | 25 µg/mL | 99.66719 | 98.63041 | 99.14880 | 0.733111 |
| **% Cell viability** | | | | | |
| **peptide** | **concentrations** | **exp. 1** | **exp. 2** | **x̄** | **sd** |
| **CYCKLK** | 0 µg/mL | 100.0000 | 100.0000 | 100.0000 | 0.00000 |
|  | 1 µg/mL | 99.60968 | 99.77885 | 99.69426 | 0.11962 |
|  | 5 µg/mL | 99.60968 | 99.85921 | 99.73445 | 0.17644 |
|  | 10 µg/mL | 99.28058 | 99.97984 | 99.63021 | 0.49445 |
|  | 25 µg/mL | 99.60846 | 99.53676 | 99.57261 | 0.05070 |

**Fig 2.** **Effects of KLK peptide and its analogs on LPS-stimulated NO production in RAW 264.7 macrophages**

|  |  | **Nitrite (µM)** | |  |  |
| --- | --- | --- | --- | --- | --- |
|  | **concentrations** | **exp. 1** | **exp. 2** | **x̄** | **sd** |
| **control** |  | 0.95955 | 0.46148 | 0.71052 | 0.35219 |
| **LPS** | 1 µg/mL | 26.52238 | 26.98606 | 26.75422 | 0.32787 |
|  |  | **Nitrite (µM)** | |  |  |
| **peptide** | **concentrations** | **exp. 1** | **exp. 2** | **x̄** | **sd** |
| **KLK** | 1 µg/mL | 1.87640 | 2.77320 | 2.32480 | 0.63413 |
|  | 5 µg/mL | 1.04444 | 1.20408 | 1.12426 | 0.11288 |
|  | 10 µg/mL | 0.71111 | 1.38776 | 1.04943 | 0.47846 |
|  | 25 µg/mL | 0.00000 | 0.00000 | 0.00000 | 0.00000 |
|  |  | **Nitrite (µM)** | |  |  |
| **peptide** | **concentrations** | **exp. 1** | **exp. 2** | **x̄** | **sd** |
| **KLK1** | 1 µg/mL | 4.57303 | 4.98969 | 4.78136 | 0.29462 |
|  | 5 µg/mL | 4.10000 | 2.78571 | 3.44286 | 0.92934 |
|  | 10 µg/mL | 3.71111 | 5.31633 | 4.51372 | 1.13506 |
|  | 25 µg/mL | 0.00000 | 0.00000 | 0.00000 | 0.00000 |
|  |  | **Nitrite (µM)** | |  |  |
| **peptide** | **concentrations** | **exp. 1** | **exp. 2** | **x̄** | **sd** |
| **KLK2** | 1 µg/mL | 7.55056 | 8.34021 | 7.94538 | 0.55836 |
|  | 5 µg/mL | 6.15556 | 6.00000 | 6.07778 | 0.10999 |
|  | 10 µg/mL | 4.82222 | 6.59184 | 5.70703 | 1.25131 |
|  | 25 µg/mL | 2.40659 | 2.44792 | 2.42726 | 0.02922 |
|  |  | **Nitrite (µM)** | |  |  |
| **peptide** | **concentrations** | **exp. 1** | **exp. 2** | **x̄** | **sd** |
| **SSKLK** | 1 µg/mL | 7.21348 | 7.87629 | 7.54489 | 0.46867 |
|  | 5 µg/mL | 5.82222 | 5.28571 | 5.55397 | 0.37937 |
|  | 10 µg/mL | 4.21111 | 4.19388 | 4.20249 | 0.01219 |
|  | 25 µg/mL | 0.64835 | 0.98958 | 0.81897 | 0.24129 |
|  |  | **Nitrite (µM)** | |  |  |
| **peptide** | **concentrations** | **exp. 1** | **exp. 2** | **x̄** | **sd** |
| **CYCKLK** | 1 µg/mL | 4.74157 | 5.96907 | 5.35532 | 0.86797 |
|  | 5 µg/mL | 3.37778 | 3.65306 | 3.51542 | 0.19465 |
|  | 10 µg/mL | 0.00110 | 0.41837 | 0.20973 | 0.29505 |
|  | 25 µg/mL | 0.00000 | 0.00000 | 0.00000 | 0.00000 |

**Fig 3.** **Effects of KLK peptide and its analogs on proinflammatory cytokine production in LPS-stimulated RAW 264.7 macrophages.**

**Fig 3A IL-1β production**

| **IL-1β (pg/mL)** | | | | | |
| --- | --- | --- | --- | --- | --- |
|  | **concentration** | **exp. 1** | **exp. 2** | **x̄** | **sd** |
| **control** |  | 49.57692 | 37.75000 | 43.66346 | 8.36290 |
| **LPS** | 1 µg/mL | 181.01923 | 177.26923 | 179.14423 | 2.65165 |
| **IL-1β (pg/mL)** | | | | | |
| **peptide** | **concentrations** | **exp. 1** | **exp. 2** | **x̄** | **sd** |
| **KLK** | 1 µg/mL | 132.46154 | 127.46154 | 129.96000 | 3.53553 |
|  | 5 µg/mL | 105.15385 | 100.15385 | 102.65380 | 3.53553 |
|  | 10 µg/mL | 65.15385 | 69.00000 | 67.07692 | 2.71964 |
|  | 25 µg/mL | 65.15385 | 60.15385 | 62.65385 | 3.53553 |
| **IL-1β (pg/mL)** | | | | | |
| **peptide** | **concentrations** | **exp. 1** | **exp. 2** | **x̄** | **sd** |
| **KLK1** | 1 µg/mL | 139.00000 | 138.23077 | 138.62000 | 0.54393 |
|  | 5 µg/mL | 119.76923 | 120.15385 | 119.96150 | 0.27196 |
|  | 10 µg/mL | 81.30769 | 93.23077 | 87.26923 | 8.43089 |
|  | 25 µg/mL | 62.07692 | 68.61538 | 65.34615 | 4.62339 |
| **IL-1β (pg/mL)** | | | | | |
| **peptide** | **concentrations** | **exp. 1** | **exp. 2** | **x̄** | **sd** |
| **KLK2** | 1 µg/mL | 163.23077 | 153.23077 | 158.23000 | 7.07107 |
|  | 5 µg/mL | 139.00000 | 130.92308 | 134.96150 | 5.71125 |
|  | 10 µg/mL | 133.61538 | 125.15385 | 129.38460 | 5.98321 |
|  | 25 µg/mL | 122.46154 | 109.00000 | 115.73080 | 9.51875 |
| **IL-1β (pg/mL)** | | | | | |
| **peptide** | **concentrations** | **exp. 1** | **exp. 2** | **x̄** | **sd** |
| **SSKLK** | 1 µg/mL | 155.53846 | 158.23077 | 156.88000 | 1.90375 |
|  | 5 µg/mL | 125.53846 | 121.30769 | 123.42310 | 2.99161 |
|  | 10 µg/mL | 114.38462 | 112.84615 | 113.61540 | 1.08786 |
|  | 25 µg/mL | 98.23077 | 95.15385 | 96.69231 | 2.17571 |
| **IL-1β (pg/mL)** | | | | | |
| **peptide** | **concentrations** | **exp. 1** | **exp. 2** | **x̄** | **sd** |
| **CYCKLK** | 1 µg/mL | 137.84615 | 131.69231 | 134.77000 | 4.35143 |
|  | 5 µg/mL | 110.15385 | 105.53846 | 107.84620 | 3.26357 |
|  | 10 µg/mL | 77.46154 | 83.61538 | 80.53846 | 4.35143 |
|  | 25 µg/mL | 63.23077 | 70.92308 | 67.07692 | 5.43928 |

**Fig. 3B TNF-α production**

|  |  | **TNF-α (pg/mL)** | |  |  |
| --- | --- | --- | --- | --- | --- |
|  | **concentrations** | **exp. 1** | **exp. 2** | **x̄** | **sd** |
| **control** |  | 18.07770 | 14.83737 | 16.45754 | 2.29126 |
| **LPS** | 1 µg/mL | 479.21397 | 476.95366 | 478.08380 | 1.59828 |
|  |  | **TNF-α (pg/mL)** | |  |  |
| **peptide** | **concentrations** | **exp. 1** | **exp. 2** | **x̄** | **sd** |
| **KLK** | 1 µg/mL | 222.79167 | 216.00000 | 219.39580 | 4.80243 |
|  | 5 µg/mL | 106.81633 | 105.46939 | 106.14290 | 0.95243 |
|  | 10 µg/mL | 78.02041 | 77.12245 | 77.57143 | 0.63495 |
|  | 25 µg/mL | 40.63265 | 35.79167 | 38.21216 | 3.42309 |
|  |  | **TNF-α (pg/mL)** | |  |  |
| **peptide** | **concentrations** | **exp. 1** | **exp. 2** | **x̄** | **sd** |
| **KLK1** | 1 µg/mL | 270.08333 | 265.59184 | 267.83760 | 3.17597 |
|  | 5 µg/mL | 138.65306 | 140.46939 | 139.56120 | 1.28434 |
|  | 10 µg/mL | 94.75510 | 93.75510 | 94.25510 | 0.70711 |
|  | 25 µg/mL | 56.85714 | 55.47917 | 56.16815 | 0.97438 |
|  |  | **TNF-α (pg/mL)** | |  |  |
| **peptide** | **concentrations** | **exp. 1** | **exp. 2** | **x̄** | **sd** |
| **KLK2** | 1 µg/mL | 291.33333 | 285.38776 | 288.36050 | 4.20416 |
|  | 5 µg/mL | 161.61224 | 163.22449 | 162.41840 | 1.14003 |
|  | 10 µg/mL | 111.08163 | 108.24490 | 109.66330 | 2.00587 |
|  | 25 µg/mL | 74.51020 | 73.60417 | 74.05719 | 0.64067 |
|  |  | **TNF-α (pg/mL)** | |  |  |
| **peptide** | **concentrations** | **exp. 1** | **exp. 2** | **x̄** | **sd** |
| **SSKLK** | 1 µg/mL | 290.60417 | 286.81633 | 288.71020 | 2.67841 |
|  | 5 µg/mL | 160.89796 | 159.44898 | 160.17350 | 1.02458 |
|  | 10 µg/mL | 98.42857 | 98.55102 | 98.48980 | 0.08659 |
|  | 25 µg/mL | 62.06122 | 59.75000 | 60.90561 | 1.63428 |
|  |  | **TNF-α (pg/mL)** | |  |  |
| **peptide** | **concentrations** | **exp. 1** | **exp. 2** | **x̄** | **sd** |
| **CYCKLK** | 1 µg/mL | 241.22917 | 241.91837 | 241.57380 | 0.48734 |
|  | 5 µg/mL | 123.75510 | 123.83673 | 123.79590 | 0.05772 |
|  | 10 µg/mL | 90.36735 | 87.93878 | 89.15306 | 1.71726 |
|  | 25 µg/mL | 52.87755 | 50.27083 | 51.57419 | 1.84323 |

**Fig 4.** **Effect of KLK peptide on LPS-induced PGE_2_ production in RAW 264.7 macrophages.**

|  |  | **PGE_2_ (pg/mL)** | | |  |
| --- | --- | --- | --- | --- | --- |
|  | **concentrations** | **exp. 1** | **exp. 2** | **x̄** | **sd** |
| **control** |  | 208.67000 | 197.71000 | 203.19000 | 7.74989 |
| **LPS** | 1 µg/mL | 11930.47000 | 11125.44500 | 11527.95750 | 569.23864 |
|  |  | **PGE_2_ (pg/mL)** | | |  |
| **peptide** | **concentrations** | **exp. 1** | **exp. 2** | **x̄** | **sd** |
| **KLK** | 5 µg/mL | 2685.16500 | 3243.62000 | 2964.39250 | 394.88732 |
|  | 10 µg/mL | 1924.59000 | 2081.93000 | 2003.26000 | 111.25618 |
|  | 25 µg/mL | 1353.88000 | 1260.32500 | 1307.10250 | 66.15337 |
|  |  | **PGE_2_ (pg/mL)** | | |  |
|  | **concentrations** | **exp. 1** | **exp. 2** | **x̄** | **sd** |
| **Indomethacin** | 10 µM | 783.51000 | 715.98500 | 749.74750 | 47.74739 |

**Fig 5.** **Effects of KLK peptide on iNOS, COX-2, IL-1β and TNF-α mRNA expression in LPS-stimulated RAW 264.7 macrophages.**

| **iNOS/GAPDH** | | | | | | |
| --- | --- | --- | --- | --- | --- | --- |
|  | **concentrations** | **exp. 1** | **exp. 2** | **exp. 3** | **x̄** | **sd** |
| **control** |  | 0.10117 | 0.12135 | 0.13629 | 0.11960 | 0.01762 |
| **LPS** | 1 µg/mL | 1.46730 | 1.47793 | 1.48416 | 1.47646 | 0.00852 |
| **KLK** | 5 µg/mL | 1.22136 | 1.14547 | 1.01435 | 1.12706 | 0.10473 |
|  | 10 µg/mL | 1.00248 | 0.96888 | 0.88202 | 0.95113 | 0.06216 |
|  | 25 µg/mL | 0.56460 | 0.36082 | 0.29422 | 0.40655 | 0.14087 |
| **COX-2/GAPDH** | | | | | | |
|  | **concentrations** | **exp. 1** | **exp. 2** | **exp. 3** | **x̄** | **sd** |
| **control** |  | 0.00019 | 0.02295 | 0.01285 | 0.01200 | 0.01140 |
| **LPS** | 1 µg/mL | 1.00991 | 1.27326 | 1.07277 | 1.11865 | 0.13754 |
| **KLK** | 5 µg/mL | 0.87771 | 0.91471 | 0.83129 | 0.87457 | 0.04180 |
|  | 10 µg/mL | 0.50938 | 0.61895 | 0.54329 | 0.55721 | 0.05609 |
|  | 25 µg/mL | 0.25148 | 0.27667 | 0.26062 | 0.26292 | 0.01275 |
| **IL-1β/GAPDH** | | | | | | |
|  | **concentrations** | **exp. 1** | **exp. 2** | **exp. 3** | **x̄** | **sd** |
| **control** |  | 0.31817 | 0.27504 | 0.28766 | 0.29362 | 0.02218 |
| **LPS** | 1 µg/mL | 1.23779 | 1.46571 | 1.35617 | 1.35322 | 0.11399 |
| **KLK** | 5 µg/mL | 1.13524 | 1.11835 | 1.03669 | 1.09676 | 0.05270 |
|  | 10 µg/mL | 0.91033 | 0.90978 | 0.85561 | 0.89191 | 0.03143 |
|  | 25 µg/mL | 0.59964 | 0.52469 | 0.59260 | 0.57231 | 0.04139 |
| **TNF-α/GAPDH** | | | | | | |
|  | **concentrations** | **exp. 1** | **exp. 2** | **exp. 3** | **x̄** | **sd** |
| **control** |  | 0.47807 | 0.28450 | 0.28148 | 0.34802 | 0.11264 |
| **LPS** | 1 µg/mL | 1.15234 | 1.25160 | 1.14097 | 1.18164 | 0.06085 |
| **KLK** | 5 µg/mL | 0.78532 | 0.77531 | 0.78604 | 0.78222 | 0.00600 |
|  | 10 µg/mL | 0.61137 | 0.66611 | 0.61902 | 0.63217 | 0.02964 |
|  | 25 µg/mL | 0.52602 | 0.48169 | 0.52725 | 0.51166 | 0.02596 |

**Fig 6.** **Effects of KLK peptide on iNOS and COX-2 protein expression in LPS-stimulated RAW 264.7 macrophages.**

| **COX-2/β-actin** | | | | | | |
| --- | --- | --- | --- | --- | --- | --- |
|  | **concentrations** | **exp. 1** | **exp. 2** | **exp. 3** | **x̄** | **sd** |
| **control** |  | 0.00732 | 0.00893 | 0.01581 | 0.01069 | 0.00451 |
| **LPS** | 1 µg/mL | 1.11842 | 0.92291 | 1.00631 | 1.01588 | 0.09810 |
| **KLK** | 5 µg/mL | 1.09670 | 0.73386 | 0.73216 | 0.85424 | 0.20998 |
|  | 10 µg/mL | 0.52863 | 0.35184 | 0.36838 | 0.41628 | 0.09764 |
|  | 25 µg/mL | 0.34777 | 0.18803 | 0.23463 | 0.25681 | 0.08215 |
| **iNOS/β-actin** | | | | | | |
|  | **concentrations** | **exp. 1** | **exp. 2** | **exp. 3** | **x̄** | **sd** |
| **control** |  | 0.00024 | 0.00072 | 0.00378 | 0.00158 | 0.00192 |
| **LPS** | 1 µg/mL | 0.60498 | 0.55741 | 0.65200 | 0.60480 | 0.04730 |
| **KLK** | 5 µg/mL | 0.43970 | 0.48336 | 0.50117 | 0.47474 | 0.03163 |
|  | 10 µg/mL | 0.02465 | 0.14276 | 0.15728 | 0.10823 | 0.07274 |
|  | 25 µg/mL | 0.02605 | 0.00316 | 0.00545 | 0.01155 | 0.01260 |

**Fig 7.** **Effects of KLK peptide on LPS-induced activation and translocation of NF-κB (A), and LPS-induced degradation and phosphorylation of IκB (B) in RAW 264.7 macrophages.**

**Fig. 7A**

| **Cytoplasmic-NF-κB p65/β-actin** | | | | | |
| --- | --- | --- | --- | --- | --- |
|  | **concentrations** | **exp. 1** | **exp. 2** | **x̄** | **sd** |
| **control** |  | 0.77110 | 0.95985 | 0.86548 | 0.13346 |
| **LPS** | 1 µg/mL | 0.06282 | 0.04046 | 0.05164 | 0.01581 |
| **KLK** | 5 µg/mL | 0.35782 | 0.20337 | 0.28059 | 0.10922 |
|  | 10 µg/mL | 0.53891 | 0.58339 | 0.56115 | 0.03145 |
|  | 25 µg/mL | 0.71051 | 0.72867 | 0.71959 | 0.01285 |
| **Nuclear-NF-κB p65/β-actin** | | | | | |
|  | **concentrations** | **exp. 1** | **exp. 2** | **x̄** | **sd** |
| **control** |  | 0.08715 | 0.11140 | 0.09927 | 0.01715 |
| **LPS** | 1 µg/mL | 0.57498 | 0.62067 | 0.59783 | 0.03231 |
| **KLK** | 5 µg/mL | 0.42533 | 0.49657 | 0.46095 | 0.05037 |
|  | 10 µg/mL | 0.28672 | 0.40969 | 0.34820 | 0.08695 |
|  | 25 µg/mL | 0.11319 | 0.28316 | 0.19817 | 0.12019 |

**Fig. 7B**

| **IκB/β-actin** | | | | | | |
| --- | --- | --- | --- | --- | --- | --- |
|  | **concentrations** | **exp. 1** | **exp. 2** | **exp. 3** | **x̄** | **sd** |
| **control** |  | 0.55670 | 0.45195 | 0.47132 | 0.49332 | 0.05573 |
| **LPS** | 1 µg/mL | 0.04807 | 0.03173 | 0.05016 | 0.04332 | 0.01009 |
| **KLK** | 5 µg/mL | 0.12157 | 0.12662 | 0.12329 | 0.12383 | 0.00256 |
|  | 10 µg/mL | 0.21825 | 0.20091 | 0.21789 | 0.21235 | 0.00991 |
|  | 25 µg/mL | 0.31217 | 0.30645 | 0.30722 | 0.30862 | 0.00311 |

| **p-IκB/β-actin** | | | | | |
| --- | --- | --- | --- | --- | --- |
|  | **concentrations** | **exp. 1** | **exp. 2** | **x̄** | **sd** |
| **control** |  | 0.07873 | 0.05615 | 0.06744 | 0.01597 |
| **LPS** | 1 µg/mL | 0.75356 | 0.74271 | 0.74814 | 0.00768 |
| **KLK** | 5 µg/mL | 0.71879 | 0.67901 | 0.69890 | 0.02813 |
|  | 10 µg/mL | 0.42459 | 0.43362 | 0.42910 | 0.00638 |
|  | 25 µg/mL | 0.08059 | 0.07921 | 0.07990 | 0.00098 |

**Fig 8.** **Binding of the KLK peptide to LPS.**

| **Nitrite (µM)** | | | | | |
| --- | --- | --- | --- | --- | --- |
| **Wash then LPS** | **concentrations** | **exp. 1** | **exp. 2** | **x̄** | **sd** |
| **LPS** | 1 µg/mL | 29.06667 | 29.74766 | 29.40717 | 0.481538 |
| **KLK** | 10 µg/mL | 9.288889 | 4.186916 | 6.737902 | 3.60764 |
| **PMB** | 10 µg/mL | 28.36296 | 29.70093 | 29.03195 | 0.946089 |
|  |  |  |  |  |  |
| **Nitrite (µM)** | | | | | |
| **No wash then LPS** | **concentrations** | **exp. 1** | **exp. 2** | **x̄** | **sd** |
| **LPS** | 1 µg/mL | 29.25185 | 28.81308 | 29.03247 | 0.310256 |
| **KLK** | 10 µg/mL | 5.140741 | 8.018692 | 6.579716 | 2.035019 |
| **PMB** | 10 µg/mL | 3.140741 | 5.775701 | 4.458221 | 1.863198 |
